# Improving plant drought tolerance and growth under water limitation through combinatorial engineering of signalling networks

**DOI:** 10.1111/pbi.13441

**Published:** 2020-07-26

**Authors:** Philipp Schulz, Katrin Piepenburg, Ruth Lintermann, Marco Herde, Mark A. Schöttler, Lena K. Schmidt, Stephanie Ruf, Jörg Kudla, Tina Romeis, Ralph Bock

**Affiliations:** ^1^ Max‐Planck‐Institut für Molekulare Pflanzenphysiologie Potsdam‐Golm Germany; ^2^ Institut für Biologie Freie Universität Berlin Berlin Germany; ^3^ Institut für Biologie und Biotechnologie der Pflanzen Westfälische Wilhelms‐Universität Münster Münster Germany; ^4^ Present address: Department of Molecular Nutrition and Biochemistry of Plants Leibniz Universität Hannover Herrenhäuser Str. 2 Hannover 30419 Germany; ^5^ Present address: Leibniz‐Institut für Pflanzenbiochemie (IPB) Weinberg 3 Halle/Saale D‐06120 Germany

**Keywords:** abiotic stress, *Arabidopsis thaliana*, drought stress, *Nicotiana tabacum*, salt stress, stress tolerance, synthetic biology, water‐use efficiency

## Abstract

Agriculture is by far the biggest water consumer on our planet, accounting for 70 per cent of all freshwater withdrawals. Climate change and a growing world population increase pressure on agriculture to use water more efficiently (‘more crop per drop’). Water‐use efficiency (WUE) and drought tolerance of crops are complex traits that are determined by many physiological processes whose interplay is not well understood. Here, we describe a combinatorial engineering approach to optimize signalling networks involved in the control of stress tolerance. Screening a large population of combinatorially transformed plant lines, we identified a combination of calcium‐dependent protein kinase genes that confers enhanced drought stress tolerance and improved growth under water‐limiting conditions. Targeted introduction of this gene combination into plants increased plant survival under drought and enhanced growth under water‐limited conditions. Our work provides an efficient strategy for engineering complex signalling networks to improve plant performance under adverse environmental conditions, which does not depend on prior understanding of network function.

## Introduction

At least 20% of all developing countries will face water shortages by 2030 (Food and Agriculture Organization of the United Nations: http://www.fao.org/english/newsroom/focus/2003/water.htm), making drought tolerance, growth under limited water availability and the efficient use of water by crop plants (and, consequently, the saving of water for irrigation) one of the highest priorities in agriculture. Water‐use efficiency (WUE) of plants refers to the ratio of water used in metabolism to water lost through transpiration and other processes. The WUE determines the plant's ability to cope with moderate or severe soil water deficit, represents a major factor in plant survival under drought stress, and is the key determinant of crop yield under conditions of limited water availability. Therefore, increasing the WUE of agricultural crops represents a major goal of breeding and genetic engineering efforts (Lawlor, 2013; Nuccio *et al*., 2015).

The mechanisms underlying drought tolerance and WUE are highly complex, and a number of physiological processes and signal transduction pathways have been suggested as potential targets to improve WUE and stress adaptation upon water limitation in vascular plants. These include the light reactions of photosynthesis (Glowacka *et al*., 2018), photosynthetic carbon fixation (Flexas *et al*., 2016), plant hormones and associated signalling pathways (Yang *et al*., 2016), stomatal function (Papanatsiou *et al*., 2019; Takahashi *et al*., 2018) and trichome density on the leaf surface (Galdon‐Armero *et al*., 2018). To improve the primary response to water limitation, modulating the perception of the stress hormone abscisic acid (ABA) appears to represent a promising strategy (Mega *et al*., 2019; Yang *et al*., 2016). In addition, numerous targets in the pathways of stress recognition and signalling are explored to increase tolerance to water limitation (Gong *et al*., 2020; Hirayama and Shinozaki, 2010). These pathways offer particularly attractive targets in that they have been implicated in natural variation in stress tolerance (Bechtold *et al*., 2018; Wu *et al*., 2012). This may be due to them exerting control over multiple downstream pathways, and/or genetic alterations in sensing and signalling having a higher probability of being phenotypically neutral (compared to constitutive manipulations of metabolic pathways or developmental processes involved in stress protection). A major challenge in exploring signalling mechanisms as targets lies in the complex organization of the underlying networks and the (partial) genetic redundancy, with network components often being encoded by large gene families and/or sharing overlapping functions.

Calcium signalling networks have emerged as major regulators of abiotic and biotic stress responses in plants (Batistic and Kudla, 2004; Hepler, 2005; Luan, 2008). Although calcium signalling represents an ancient signalling mechanism in eukaryotes, the protein families involved in decoding calcium signals have undergone substantial expansion during evolution of the green lineage (Hrabak *et al*., 2003; Kolukisaoglu *et al*., 2004). One of the major protein families mediating responses to stress‐induced changes in intracellular calcium concentration are the calcium‐dependent protein kinases (CDPKs or, in *Arabidopsis*, CPKs), in which a protein kinase effector domain is directly linked with a calcium‐binding sensor domain within one molecule. The family comprises 34 members in the model plant *Arabidopsis thaliana* (Cheng *et al*., 2002; Edel *et al*., 2017). CDPK family members have been implicated in diverse biological functions, including long‐term growth and developmental processes as well as short‐term rapid signalling responses to abiotic and biotic stress stimuli (Brandt *et al*., 2015; Edel *et al*., 2017; Huang *et al*., 2018; Liu *et al*., 2017; Schulz *et al*., 2013). Furthermore, within the calcium signalling network, evidence for joint or consecutive action of CPKs or concerted action with other calcium sensor protein kinases (Maierhofer *et al*., 2014; Zhao *et al*., 2013) on specific target proteins in the plasma membrane has been obtained. The large number of CPKs, the complex structure of the signalling network they form (including potential functional interconnection and synergistic action of family members) and the great diversity of physiological responses CPK family members are involved in (Cheng *et al*., 2002; Edel *et al*., 2017; Schulz *et al*., 2013) suggests that the engineering of individual network components may have limited (durable) effects on stress tolerance and yield stability. Unfortunately, next to nothing is known about the functional interconnection and synergistic action of the family members. We, therefore, decided to explore a combinatorial approach based on synthetic network selection towards the identification of gene combinations that enhance stress tolerance, using drought tolerance and plant growth under water limitation as biological readout. To this end, we employed combinatorial genetic transformation, a large‐scale co‐transformation technology that has successfully been applied to metabolic pathway engineering in plants (Zhu *et al*., 2008; Naqvi, Farré et al., 2009; Naqvi, Zhu et al., 2019; Fuentes *et al*., 2016; reviewed, e.g. in Naqvi, Farré et al.,^2009^; Naqvi, Zhu et al., 2019, Bock, 2013). We reasoned that combinatorial transformation should also provide a suitable approach to engineer complex signalling networks and, thus, significantly expand our available toolbox for the engineering of crops for improved stress tolerance. Its application to calcium signalling provides the potential advantage of maintaining spatial and temporal control of kinase activities through the plant's own calcium signals as triggered by environmental stimuli.

## Results

### Combinatorial genetic transformation of tobacco plants with a large set of *CPK* genes

**Figure 1:**
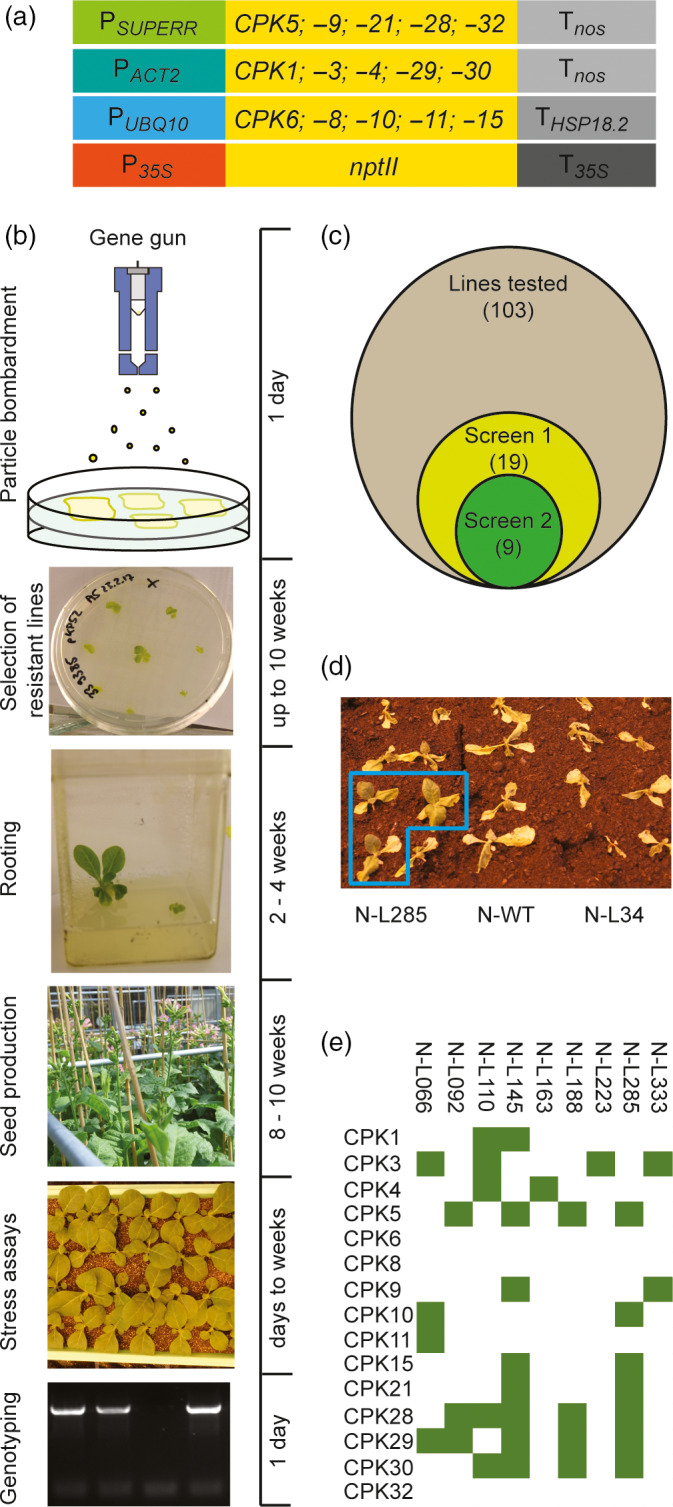
Combinatorial transformation and screening for drought tolerance. (a) Schematic representation of the vectors used for combinatorial transformation of tobacco. See text and Methods for details. (b) Workflow of the combinatorial transformation experiment, screening and genotyping. The approximate timeline is given at the right. (c) Diagram showing the number of combinatorially transformed lines assayed (103), the number of drought‐tolerant lines identified in the first screening experiment (19), and the final number of confirmed tolerant lines (9). (d) Identification of a combinatorially transformed line (N‐L285) that segregates drought‐tolerant plants in the T1 generation. (e) Transgene contents of the nine drought‐tolerant lines, as determined by PCR assays.

Combinatorial transformation relies on the generation of a large library of transgenic plants carrying random combinations of transgenes that are cloned into individual plasmids and co‐transformed biolistically (Bock, 2013; Naqvi, Farré et al.,^2009^; Naqvi, Zhu et al.,^2019^). Combinatorial transformation experiments require highly efficient biolistic transformation protocols, making tobacco (*Nicotiana tabacum*) a suitable host species (Fuentes *et al*., 2016). However, it needs to be borne in mind that, when ten or more transgenes are used, only a small part of the combinatorial space can be explored, given that, in a simplified manner, the total space can be considered as a combination with repetition problem (with n being the total number of transgenes and k any possible number of transgenes that integrate, with the additional complication that not all k are equally likely). Initiation and propagation of stress‐related calcium signalling in plants often occur at the plasma membrane. The calcium sensor proteins involved typically carry N‐terminal lipid modifications (myristoylation and/or palmitoylation) that anchor them to the membrane (Cheng *et al*., 2002; Held *et al*., 2011; Schulz *et al*., 2013). We, therefore, selected CPKs from *Arabidopsis* that are predicted to be membrane associated (according to the SUBA database; Heazlewood *et al*., 2007). We used *Arabidopsis* because, when the project was initiated, no draft genome of *Nicotiana tabacum* was available, making gene identification in this allotetraploid species very challenging (Sierro *et al*., 2014). The corresponding 15 *CPK* genes (Figure 1a) were cloned as cDNAs into individual expression cassettes using three different strong, constitutive promoters (*SUPERR*, *ACT2*, *UBQ10*) and two different terminators (*nos*, *HSP18.2*) to minimize potential silencing effects. In this way, 15 plasmids were generated and combined with an additional plasmid that harboured an *nptII* cassette for kanamycin selection of transgenic lines. The mix of 16 plasmids was loaded onto gold particles and bombarded into tobacco cells (Figure 1b).

Combinatorial transformation experiments followed by large‐scale selection of transgenic lines produced more than 450 primary kanamycin‐resistant lines. As from an agricultural perspective, increased stress tolerance should come without a growth penalty, lines displaying atypical growth and/or development were discarded and not further analysed (even though this may have eliminated some events with particularly pronounced effects on stress tolerance). 231 lines readily developed roots on phytohormone‐free medium, 206 of which displayed normal growth in sterile culture and were transferred to the greenhouse for seed production. Upon growth in the greenhouse, 52 lines showed growth impairment and/or developmental aberrations and were discarded. Another 51 lines exhibited low fertility and/or poor seed production and were also excluded from further analysis. The remaining 103 lines displayed wild type‐like growth and seed set, and the T1 seeds harvested from them were used in subsequent screens for elevated drought tolerance (Figure 1b,c).

### Screening of the population of combinatorially transformed plants for drought tolerance

In an initial high‐throughput screen for combinatorially transformed lines with enhanced drought tolerance, batches of 40 plants were grown together in one tray to ensure that the drought stress sets in synchronously and affects all plants equally. To this end, two transgenic lines (16 T1 individuals each) were grown together with eight wild‐type plants. It is important to note that the T1 generation represents the progeny of the primary transgenic lines which are hemizygous for the transgenes they contain. Consequently, the T1 generation will typically segregate into transgene‐containing and transgene‐free individuals in a 3:1 ratio (assuming that all transgenes integrated into a single genomic locus, which is usually the case in combinatorial transformation; Bock, 2013; Fuentes *et al*., 2016; Naqvi, Farré et al.,^2009^; Naqvi, Zhu et al.,^2019^; Zhu *et al*., 2008).

To screen for drought tolerance, water was withheld for 23 days, and, 7 days after re‐watering, the number of surviving individuals was counted. In this initial screen, 19 lines displayed enhanced drought tolerance (assayed as increased survivorship; Figure 1c,d). To eliminate false positives, the screen was repeated with a larger number of individuals (now growing only one transgenic line and the wild type together in one tray). This second screening experiment confirmed the drought tolerance of nine of the 19 lines that had been identified in the initial screen (Figure 1c). The nine confirmed tolerant lines were then genotyped by PCR and assayed for the presence of the 15 *CPK* transgenes (by amplification of their full‐length coding regions). The transgene sets detected in the drought‐tolerant lines (Figure 1e) were then inspected to identify genes and gene combinations that were frequently represented in the lines.

*CPK3* and *CPK30* were identified in four lines each (Figure 1e). Their presence was not unexpected, as both genes had been associated with ABA signalling and stomatal closure previously (Mori *et al*., 2006; Sheen, 1996). Somewhat unexpectedly, *CPK5*, a gene previously associated with biotic stress tolerance, also appeared in four lines (Boudsocq *et al*., 2010; Dubiella *et al*., 2013). The two most abundant transgenes present in the drought‐tolerant lines were *CPK28* and *CPK29*, both of which were found five times. Moreover, the two genes co‐occurred in four of the five lines. Neither *CPK28* nor *CPK29* have been implicated in abiotic stress responses so far. While *CPK29* has not yet been functionally characterized, *CPK28* was shown to participate in balancing innate immune signalling, and jasmonic acid and gibberellic acid homeostasis during development (e.g. Matschi *et al*., 2015; Matschi *et al*., 2013; Monaghan *et al*., 2014).

To exclude possible biases in the representation of the individual *CPK* transgenes in the population of combinatorially transformed lines, the presence of two transgenes that had been identified as potentially overrepresented in the drought‐tolerant lines (Figure 1e) was exemplarily assayed in the entire population (Table S1). Both transgenes (*CPK3* and *CPK29*) were present in approximately 20% of the 103 lines (Figure 1c; Table S1), indicating that their abundance in the population reflects the 1:1 stoichiometry of the plasmids used for combinatorial transformation. By contrast, *CPK3* was present in 44.4% and CPK29 in 55.5% of the drought‐tolerant lines, suggesting that the two transgenes had been selected for in our screen for drought tolerance. However, it should be noted that a somewhat uneven representation of *CPK* genes in the population of transgenic lines is to be expected, because lines showing impaired growth or development were discarded (see above) or may not even be recoverable as transgenic events, thus potentially leading to an underrepresentation of some genes and/or gene combinations. This is exemplified by the fact that, for example, *CPK6*, *CPK8* and *CPK32* are found not even once in the drought‐tolerant lines, possibly suggesting that they were selected against (Figure 1e).

### The combination of *CPK28* and *CPK2*9 is required to enhance drought stress tolerance in *Arabidopsis*

The identification of *CPK28* and *CPK29* as the two most frequently occurring transgenes in the drought‐tolerant combinatorially transformed lines and their co‐occurrence in most lines (Figure 1e) prompted us to test whether these two genes, individually or collectively, can confer increased drought tolerance. CPK28 and CPK29 proteins share the conserved CDPK domain structure (Figure S1A) and show a partial overlap in their gene expression patterns during plant development (Figure S1B,C).

**Figure 2:**
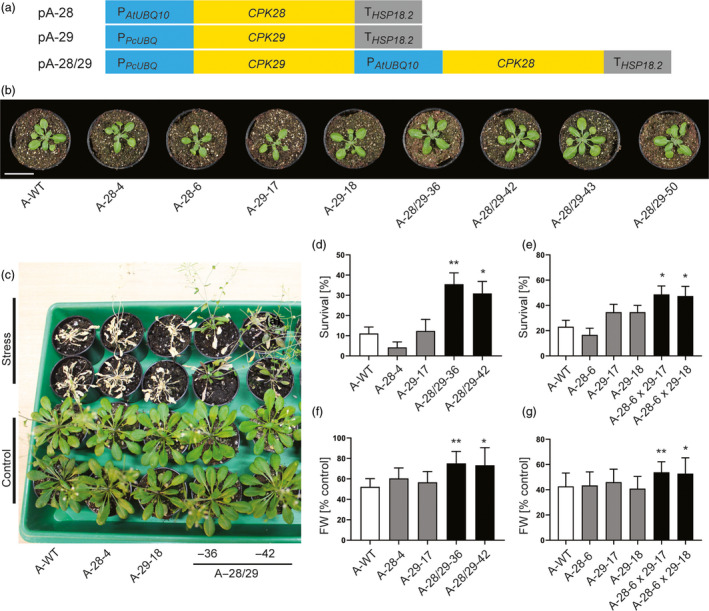
The combinatorial module pA‐28/29 confers drought tolerance to the model plant *Arabidopsis*. (a) Schematic representation of the vectors pA‐28, pA‐29 and pA‐28/29 used for stable transformation of *Arabidopsis thaliana*. (b) Representative images of transgenic *Arabidopsis* plants (A‐28, A‐29 and A‐28/29 lines) grown in soil in comparison with the wild type (A‐WT). Scale bar: 3 cm. (c) Identification of *Arabidopsis* plants that survive severe drought stress and show recovery after rewatering. Control plants were not exposed to drought stress. (d) Increased survival rate of transgenic lines produced by transformation with vector pA‐28/29. (e) Enhanced survival of doubly transgenic lines obtained from crosses of A‐28 with A‐29 lines (A‐28 × 29). (f) Increased biomass production of A‐28/29 lines grown under mild osmotic stress (in the presence of 35 mm mannitol). (g) Increased biomass production of A‐28 × 29 lines grown under mild osmotic stress. Asterisks indicate statistically significant differences between transgenic lines and the wild type as determined by one‐way ANOVA followed by Dunnett's multiple comparison (*P < 0.05, **P < 0.01). All data are presented as means ± SD from three independent experiments (*d*: *n* = 12 biological replicates, with 9 plants per line in each replicate; *e*: *n* = 26 biological replicates, with 3 plants per line in each replicate; *f*: *n* = 12 biological replicates, with 10 plants per line and condition in each replicate; and *g*: *n* = 20 biological replicates, with 10 plants per line and condition in each replicate).

To test whether each of the two genes confers drought tolerance or the combination of CPK28 and CPK29 is necessary (and sufficient), three vectors for plant transformation were generated (Figure 2a): two plasmids for individual expression of *CPK28* (vector pA‐28) and *CPK29* (vector pA‐29) and one for the combined expression of *CPK28* and *CPK29* (vector pA‐28/29). The constructs were introduced into *Arabidopsis* plants by stable *Agrobacterium*‐mediated transformation, and transgenic lines (subsequently referred to as A‐28, A‐29 and A‐28/29 lines, respectively) were isolated. 54 independent A‐28/29 lines, 26 A‐29 lines and 27 A‐28 lines were obtained, and all of them grew normally, were phenotypically inconspicuous and indistinguishable from the wild type (Figure 2b). This is in contrast to CPKs involved in biotic stress responses, whose overexpression often results in mutant phenotypes such as stunted growth and/or formation of necrotic lesions (Dubiella *et al*., 2013; Durian *et al*., 2020) as a consequence of enhanced and prolonged stress signalling.

Next, we conducted drought tolerance assays with homozygous transgenic lines by exposing plants to severe drought stress followed by re‐watering (Figure 2c). Remarkably, A‐28/29 lines showed a significant increase in survival, whereas the single gene overexpressing lines A‐28 and A‐29 were not significantly different from the wild type (Figure 2c,d; Figure S2A). This result clearly demonstrates the functional significance of the co‐occurrence of the two genes seen in our screen of the combinatorially transformed population for drought tolerance.

To further verify that CPK28 and CPK29 must be expressed together to confer drought tolerance, we crossed A‐28 lines with A‐29 lines and tested the doubly transgenic offspring (harbouring both genes) in drought tolerance assays. These analyses revealed that A‐28 x 29 lines exhibit a significantly higher survival rate compared to the wild type and the A‐28 and A‐29 parental lines (Figure 2e; Figure S2B). To mimic mild drought conditions as plants often experience them under field conditions (Skirycz *et al*., 2011), we challenged our transgenic lines with moderate osmotic stress. To this end, seeds were germinated in the presence of 35 mm mannitol (a concentration exerting osmotic stress without causing a delay in germination) and plant biomass (fresh weight) was measured after 14 days of growth. Under these conditions, both the A‐28/29 and the A‐28 x 29 lines outperformed the wild type and the A‐28 and A‐29 parental lines in that they displayed substantially larger biomass production. Importantly, the unstressed control plants showed no difference in growth (Figure 2f,g; Figures S2C‐E and S3), indicating that transgene expression does not cause a growth penalty under normal conditions.

### The *CPK28/29* module is transferable to a high‐biomass crop

**Figure 3:**
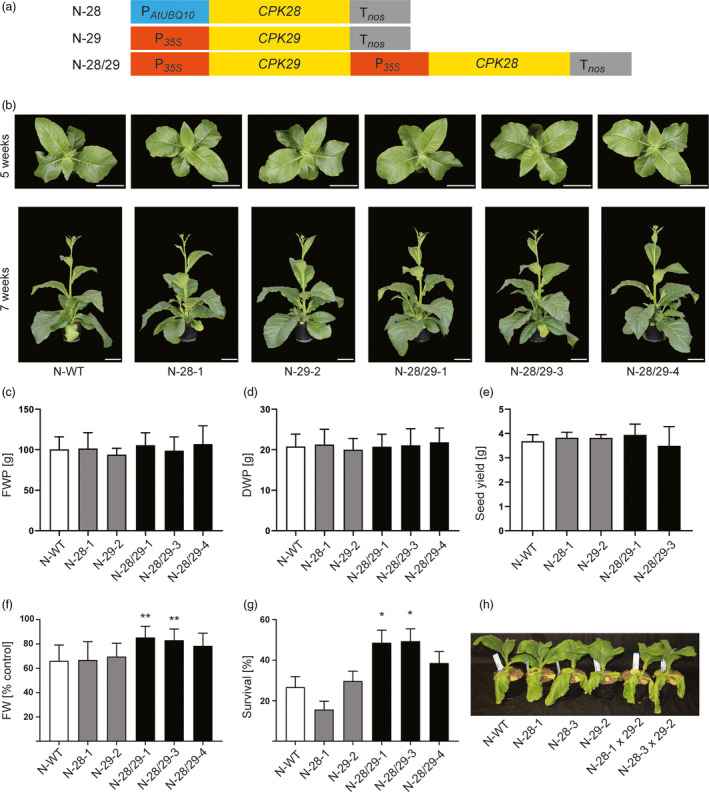
The CPK28/CPK29 module confers stress tolerance without causing a growth penalty in the high‐biomass crop tobacco. (a) Schematic overview of vectors used for transformation of tobacco. (b) Representative photographs of tobacco plants after five weeks (top view) and after seven weeks (side view) of growth. Scale bars: 10 cm. (c) Biomass production under control conditions. Leaf biomass (FWP: fresh weight per plant) was measured after 7 weeks of growth. (d) Determination of the dry weight (DWP) of the same material. (e) Seed yield. (f) Biomass production (FW) under osmotic stress conditions relative to the unstressed control. (g) Survival rate under drought stress. (h) Representative image of a wild‐type plant, two N‐28 × 29 lines, and the two parental lines (N‐28 and N‐29) after one week without watering. Asterisks indicate statistically significant differences between transgenic lines and the wild type as determined by one‐way ANOVA followed by Dunnett's multiple comparison (*P < 0.05, **P < 0.01). All data are presented as means ± SD from two (*c–e*) or three (*f, g*) independent experiments (*c–e*: *n* = 5‐6 biological replicates, each with 10 plants per line; *f*: *n* = 9 biological replicates, each with 10 plants per line; and *g*: *n* = 36 biological replicates, each with 3 plants per line).

Having obtained proof of concept in *Arabidopsis*, we next sought to demonstrate that the combined expression of CPK28 and CPK29 is necessary and sufficient to mediate stress tolerance in a high‐biomass crop. To this end, we created three constructs for stable transformation of tobacco to express CPK28 or CPK29 alone, or co‐express both genes (Figure 3a). Because initial transformation experiments with constructs based on the ubiquitin promoter were successful only for one of the constructs, the promoter was exchanged by the CaMV 35S promoter in two of the three vectors (Figure 3a). The resulting transgenic lines will subsequently be referred to as N‐28, N‐29 and N‐28/29 lines, respectively. 19 independent N‐28 lines, 16 N‐29 lines and 6 N‐28/29 lines were obtained. Similar to the transgenic lines generated in the model plant *Arabidopsis* (Figure 2), no growth difference or phenotypic aberrations were seen upon plant cultivation under standard conditions in any of the transgenic lines (Figure 3b). Neither plant biomass (determined as both fresh weight and dry weight; Figure 3c,d) nor total seed yield (Figure 3e) were different between any of the transgenic lines and the wild‐type control.

To assay the transgenic lines for stress tolerance, we first performed experiments under low (mannitol‐induced) osmotic stress. The data revealed that the N‐28/29‐1 and N28/29‐3 lines produced substantially more leaf biomass (measures as fresh weight) under these conditions than the wild type or any of the single‐gene transformants (N‐28 and N‐29 lines; Figure 3f). When tested in drought tolerance assays, the N‐28/29 lines showed improved survival (Figure 3g), confirming the results obtained with our A‐28/29 transgenic *Arabidopsis* lines (Figure 2).

As an additional abiotic stress, we also determined biomass accumulation under salt stress. The N‐28/29 lines displayed enhanced germination and increased growth under these conditions (Figure S4), suggesting that the beneficial effect of the co‐expression of *CPK28* and *CPK29* may extend also to other abiotic stresses that have an osmotic component, although some of those stresses (e.g. cold) have not yet been investigated.

To ultimately confirm that the combined expression of CPK28 and CPK29 is necessary and sufficient to enhance stress resistance, N‐28 and N‐29 lines (both of which showed no elevated stress tolerance; Figure 3) were crossed. To obtain a segregating progeny, hemizygous transgenic lines were used for the crosses, resulting in a 1:4 segregation for the presence of both genes in individuals of the next generation. This approach allowed us to test if individuals containing both genes are enriched among the F1 plants that survive severe drought stress. To this end, the progeny of four crosses between N‐28 and N‐29 lines (using three different mother and two different father plants) was exposed to drought stress. No difference was observed between the parental plants and the progeny of the crosses in the initial phase of stress application (Figure 3h). PCR genotyping of the N‐28 x 29 progeny that survived the extended drought stress revealed that both transgenes were jointly present in 33‐46% of the plants, as opposed to the 25% expected by chance (Table S2). Control assays for inheritance of the kanamycin resistance confirmed that transgene inheritance was Mendelian and no unexpected segregation distortion occurred.

### CPK28/29 improves plant growth under water‐limited conditions

Having shown that, in both *Arabidopsis* and tobacco, combined expression of CPK28 and CPK29 promotes growth under mild osmotic stress conditions and enhances survival upon severe drought, we next asked if expression of the CPK28/29 module translates into increased plant productivity upon long‐term growth under water‐limited conditions. Tolerance to moderate water deficit is an important agronomic trait and often represents the limiting factor that determines crop yield under field conditions (Skirycz *et al*., 2011).

**Figure 4:**
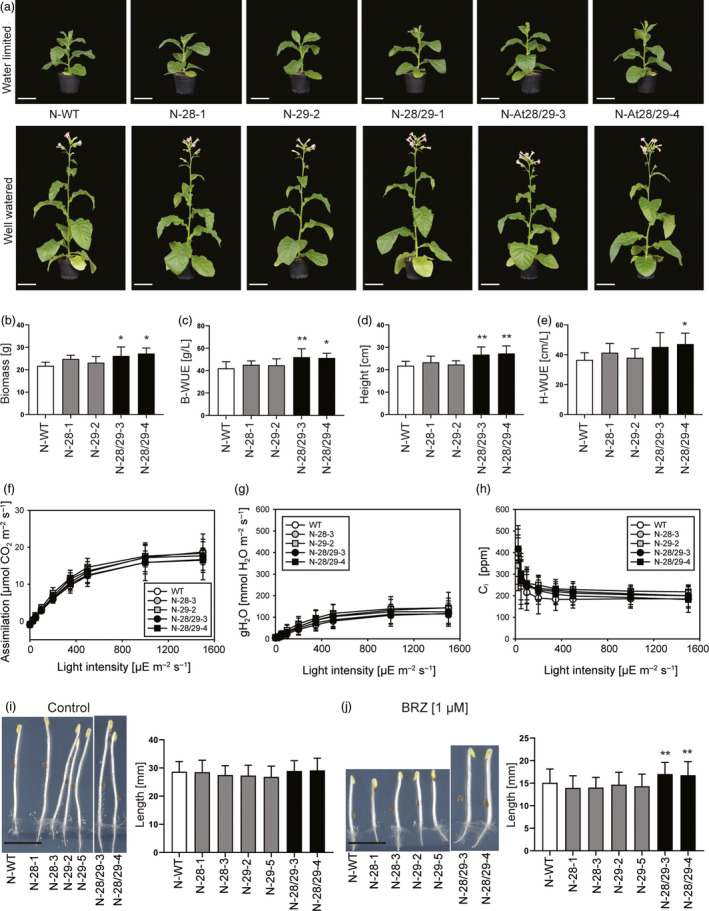
Improved growth and WUE of N‐28/29 lines under water‐limited conditions. (a) Representative pictures of eight‐week‐old tobacco plants grown under restricted watering (top panel) or well‐watered conditions (lower panel). Scale bars: 10 cm. (b) Biomass (fresh weight) of plants after 7 weeks of growth. (c) WUE of biomass production (total leaf biomass in gram per litre water supplied after the shift to restricted watering). (d) Plant height after 6.5 weeks of growth. (e) WUE of plant height reached (total height in cm per litre water supplied after the shift to restricted watering). (f) Measurement of photosynthetic assimilation capacity under water‐limited conditions. (g) Measurement of stomatal conductance. (h) Measurement of the internal CO_2_ concentration in leaves. N‐WT: wild‐type tobacco plants. No statistically significant differences can be detected between lines (determined by one‐way ANOVA). For a plot of the intrinsic water‐use efficiency (iWUE), see Figure S5. (i, j) N‐28/29 plants display reduced sensitive to BRZ treatment. Seedlings were grown seven days in the dark without (i) or with (j) 1 µm BRZ, and hypocotyl length was measured. Scale bars: 1 cm. Asterisks indicate statistically significant differences between transgenic lines and the wild type as determined by one‐way ANOVA followed by Dunnett's multiple comparison (*P < 0.05, **P < 0.01). All data presented are means ± SD with *n* = 6–7 biological replicates in (*b–e*) and n = 8 biological replicates in (*e–h*). Data shown in (*i,j*) are from one experiment, which was repeated 4 times with similar results (with *n* = 52–55 seedlings per condition).

To investigate performance under moderate water‐limiting conditions, tobacco plants were raised in normal (well‐watered) conditions for 25 days and then split into a control group and a low‐water input group (in which water supply was reduced to 25%). Confirming our previous analyses (Figures 2 and 3), no difference in plant growth was detectable in well‐watered control conditions (Figure 4a). As expected, the low‐water input group showed overall reduced growth of all lines. However, the N‐28/29 lines exhibited superior growth compared to the wild‐type control and the N‐28 and N‐29 single‐gene transformants (Figure 4a). To quantify the growth advantage of the N‐28/29 plants, a number of parameters related to biomass production and WUE were measured. N‐28/29 plants produced significantly more biomass (fresh weight) under water‐limited conditions (Figure 4b) and showed enhanced WUE in that they accumulated substantially more biomass per water supplied (Figure 4c). Moreover, N‐28/29 plants were taller than all control lines, confirming that joint expression of *CPK28* and *CPK29* enables faster growth under water‐limited conditions (Figure 4a,d,e).

To examine whether improved growth under water limitation is related to increased photosynthetic capacity, we determined light response curves of gas exchange parameters such as assimilation capacity, stomatal conductance, and CO_2_ concentration in the intercellular air space (c_i_) in both water‐limited and control conditions. Interestingly, despite their growth advantage, the N‐28/29 plants are very similar to all control plants (wild type, N‐28 and N‐29 lines) in all parameters measured (Figure 4f–h; Figure S5). In line with the unaltered gas exchange, stomatal response to the stress hormone abscisic acid (ABA) and stomatal density were similar in all genotypes (Figures S6 and S7). Likewise, key photosynthetic parameters such as chlorophyll a:b ratio, chlorophyll content, leaf absorptance, the maximum quantum efficiency of photosystem II in the dark‐adapted state, and the light‐saturated electron transport capacity of photosystem II (Figure S8) were unaltered in the transgenic lines.

In *Arabidopsis*, overexpression of the brassinosteroid receptor BRL3 (Fàbregas *et al*., 2018) has been reported to confer increased tolerance to drought stress. Since CPK28 has been implicated in the turnover of the cytoplasmic receptor‐like kinase BIK1 (BOTRYTIS‐INDUCED KINASE1; Monaghan *et al*., 2014), and *bik1* mutants display reduced sensitivity to brassinazole (BRZ), a specific inhibitor of brassinosteroid biosynthesis (Lin *et al*., 2013), it seemed reasonable to test whether co‐expression of *CPK28* and *CPK29* enhances brassinosteroid signalling, and whether this in turn supports growth under water‐limiting growth conditions. We, therefore, performed hypocotyl growth experiments in the presence of the brassinosteroid biosynthesis inhibitor BRZ. Although CPK28 overexpression has been reported to reduce BIK1 accumulation (Monaghan *et al*., 2014), no differences in hypocotyl growth were observed in the N‐28 lines compared to the wild type. However, the combined expression of *CPK28* and *CPK29* led to a substantially enhanced growth response (Figure 4i,j). This finding indicates that N‐28/29 plants are less sensitive to BRZ and suggests that co‐expression of the two calcium‐dependent kinases stimulates brassinosteroid signalling and, in this way, increases drought tolerance and growth under water‐limited conditions.

Finally, we wanted to confirm that the introduced *CPK28* and *CPK29* transgenes in our transgenic *Arabidopsis* and tobacco lines are indeed expressed and, therefore, can be causally responsible for the observed stress tolerance phenotypes. qRT‐PCR assays revealed strong expression of all transgenes, as expected (Figures S9 and S10).

### Identification and validation of a new candidate gene for improved salt tolerance

**Figure 5:**
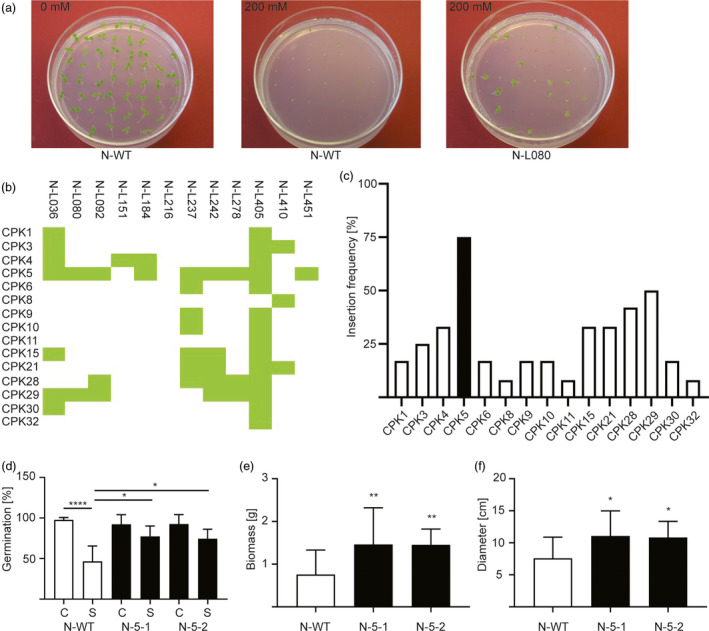
Screening of the combinatorially transformed population for salt tolerance and validation by targeted retransformation. (a) Example of a positively selected combinatorial line (N‐L080) that segregates salt‐tolerant plants in the T1 generation on synthetic medium containing 200 mm NaCl. (b) Transgene contents of the twelve salt‐tolerant lines, as determined by PCR assays. (c) Representation of each *CPK* transgene in the salt‐tolerant combinatorially transformed lines. The most frequently detected CPK (*CPK5*) is shown as black bar. (d) Seed germination rates in control conditions (C) and under salt stress (S; 200 mm NaCl). Germination was scored after nine days for the wild type and two independently generated transgenic tobacco lines transformed with a *CPK5* overexpression construct (N‐5‐1 and N‐5‐2). (e) Biomass (fresh weight per plant) of wild‐type plants, N‐5‐1 plants and N‐5‐2 plants grown in soil for one week after watering with 200 mm NaCl. (f) Plant diameter of the same plants measured directly before harvest. Asterisks indicate statistically significant differences between transgenic and wild‐type plants as determined by one‐way ANOVA and Tukey's (*d*) or Dunnett's (*e, f*) multiple comparison (*P < 0.05, **P < 0.01, ***P < 0.001). All data are presented as means ± SD from three independent experiments (*d*: *n* = 6 biological replicates with 20 seeds per line in each replicate; *e, f*: *n* = 14–16 biological replicates).

To demonstrate the general applicability of the combinatorial approach towards disentangling complex signalling networks and functionally dissecting large gene families, we screened the combinatorial *CPK* population for salt tolerance as another abiotic stress that is of great agronomic relevance. The screen was conducted by germinating seeds in the presence of 200 mm NaCl, a salt concentration that reduces germination (scored as appearance of green cotyledons) of wild‐type tobacco to nearly zero (Figure 5a). Combinatorially transformed lines that displayed an enhanced germination rate in the T1 generation (Figure 5a) were considered candidate lines for exhibiting elevated salt tolerance. 12 lines robustly showed enhanced germination in repeated tests. The 12 tolerant lines were then genotyped by PCR and assayed for the presence of the 15 *CPK* transgenes (Figure 5b). For unknown reasons, one of the lines (line N‐L216) did not contain an amplifiable *CPK* transgene (but was transgenic and harboured the kanamycin resistance gene). Interestingly, both *CPK28* and *CPK29* were found frequently in the salt‐tolerant lines, confirming the results from our assays for osmotic and salt stress tolerance conducted with retransformed *Arabidopsis* and tobacco lines (Figures 2f,g and 3F; Figures S2C‐E and S4). However, the by far most strongly represented transgene in the salt‐tolerant lines was *CPK5* (Figure 5c). To test whether *CPK5* represents a novel gene that is involved in conferring salt stress tolerance, we re‐transformed tobacco plants with a *CPK5* expression construct (see Methods). The transgenic lines were then tested for their tolerance to salt stress by measuring seed germination rates (Figure 5d), biomass (Figure 5e) and growth (plant diameter; Figure 5f) in the presence of NaCl. By all three parameters, the *CPK5*‐expressing transgenic lines performed significantly better than the control plants, confirming that CPK5 is a useful target for engineering tolerance to salt stress. To ultimately confirm expression of the *CPK5* transgene, qRT‐PCR analyses were conducted and revealed high levels of *CPK5* mRNA accumulation, as expected (Figure S11).

## Discussion

In the face of climate change and scarce water resources in many regions of the world, increasing abiotic stress tolerance and the WUE of crops is becoming one of the most pressing challenges to address through breeding and genetic engineering. In this work, we have used drought tolerance and plant growth under water‐limited conditions as model traits to combinatorially engineer a complex signal transduction network whose organization is only poorly understood. While, in previous research, some information has been obtained about the functions of individual members of the CPK protein family in *Arabidopsis* (e.g. Boudsocq *et al*., 2010; Brandt *et al*., 2015; Cheng *et al*., 2002; Dubiella *et al*., 2013; Liu *et al*., 2017; Matschi *et al*., 2015; Mori *et al*., 2006; Schulz *et al*., 2013), little is known about their functional interconnection and possible synergistic action in signalling processes. We used the CPK family of calcium‐dependent kinases to test the idea that modules of two or more *CPK* genes confer novel phenotypes that cannot be uncovered by expressing individual family members.

The gene combination identified here as enhancing drought tolerance while maintaining normal growth under unstressed conditions could neither have been predicted nor identified based on functional analysis or overexpression of single genes. Mechanistically, one could envision that both enzymes target the same protein at different phosphorylation sites, or act consecutively in that one CPK directly phosphorylates the other (and, in this way, activates it), or the two CPKs target different proteins which then synergistically results in enhanced stress tolerance. These possible mechanisms illustrate the power of our synthetic combinatorial approach in that it requires no prior information about network function.

Instead, the combinatorial gene space is explored by generating a population of transgenic lines that harbour random combinations of genes for network members, while maintaining normal growth and development. This population is then screened for the trait of interest, and individuals with the desired properties are genotyped to identify transgene combinations that are enriched and frequently co‐occur. These gene combinations are finally verified by targeted transformation experiments to confirm that they are necessary and sufficient to confer the desired trait. In this work, we have used two independent verification methods: (i) plant transformation with a vector harbouring both *CPK28* and *CPK29* expression cassettes, and (ii) plant transformation with individual cassettes followed by combination of the two cassettes in the same plant through crosses. Both approaches gave very similar results (and were confirmed in two different species), suggesting that it will be sufficient to use one of them in future applications of the technology.

Our data obtained from the extensive phenotypic characterization of retransformed plant lines (obtained by either co‐introduction of both transgenes by transformation or their combination by genetic crosses) demonstrated that CPK28 and CPK29 are jointly required to promote plant growth under water‐limited conditions and enhance plant survival under severe drought stress (Figures 2, 3, 4; Figures S2–S4). Consistent with the conservation of the CPK network across seed plants (Hrabak *et al*., 2003), the beneficial effect was seen in both the model plant *Arabidopsis* and the high‐biomass crop tobacco, suggesting that it is likely transferable also to other crops. Importantly, the enhanced biomass production under stress conditions did not entail a growth penalty under unstressed conditions (Figures 2, 3, 4; Figures S5–S8), representing a rare outcome of genetic engineering for stress tolerance in plants.

We observed reduced sensitivity of CPK28/29 lines to BRZ, possibly suggesting enhanced BR signalling. BR signalling is often seen as antagonist to ABA‐mediated responses. However, the interplay of BR signalling with abiotic stress is highly complex (Nolan *et al*., 2020). While some gain‐of‐function mutants in the pathway reduce stress tolerance, cell type‐specific activity of other BR signalling components (such as BRL3 in vascular tissue) promotes drought tolerance (Fàbregas *et al*., 2018). While it seems possible that differential phosphorylation of a common target of CPK28/29 leads to a split of stress‐related and growth‐regulatory functions (similar to the case of BES1; Kang *et al*., 2015; Nolan *et al*., 2017), this and other possible connections between calcium signalling, BR signalling and stress tolerance remain to be investigated.

It is important to note that, in combinatorial transformation, all transgenes usually integrate into the same genomic locus (i.e. into the same transient DNA double‐strand break), resulting in co‐segregation in subsequent generations (Naqvi, Farré et al.,^2009^; Naqvi, Zhu et al.,^2019^). This feature allowed us to raise a T1 generation from seeds and efficiently screen it for drought tolerance. Since tobacco produces large amounts of seeds that can be stored for many decades, the same population can be screened for other agronomic traits that may benefit from engineered calcium signalling.

Combinatorial transformation has previously been used as a tool to introduce new metabolic pathways into plants (Fuentes *et al*., 2016; Naqvi, Farré et al.,^2009^; Naqvi, Zhu et al.,^2019^; Zhu *et al*., 2008). Our data reported here suggest that it also provides a powerful approach to dissect complex signal transduction networks and engineer them to improve plant performance under adverse environmental conditions. The approach should be applicable to any signalling pathway with its known and suspected players. Importantly, the technology does not require any *a priori* knowledge about network structure and function. Instead, it can uncover hidden synergistic interactions between genes in complex genetic networks. The method can be applied to any other signal transduction network and/or response pathway involved in plant tolerance to abiotic or biotic stresses, and thus offers great potential to contribute to crop protection and the increase in agricultural productivity that is so urgently needed.

## Experimental procedures

### Plant material and transformation methods

*Arabidopsis thaliana* ecotype Col‐0 (A–WT) and *Nicotiana tabacum* cv. Petit Havana (N–WT) served as wild types in all experiments. An *Arabidopsis cpk28* mutant (*cpk28‐1*, GK‐523B08) was characterized previously (Matschi *et al*., 2015). A *cpk29* mutant was ordered from NASC (Salk‐114657C).

For combinatorial transformation, young leaves of *Nicotiana tabacum* plants grown under aseptic conditions were bombarded with gold particles that had been coated with a near equimolar plasmid mix containing the 15 individual CPK vectors (Figure 1) and an additional plasmid harbouring an *nptII* cassette as selectable marker (Fuentes *et al*., 2016). Biolistic transformation experiments were carried out with the DuPont PDS1000He gun and kanamycin‐resistant shoots were selected on an MS‐based plant regeneration medium (Murashige and Skoog, 1962) containing 50 mg/L kanamycin. Resistant shoots were rooted on phytohormone‐free medium supplemented with 50 mg/L kanamycin, then transferred to soil and grown to maturity under standard greenhouse conditions. Combinatorially transformed lines are designated N‐L followed by a consecutive number.

For re‐transformation of selected genes and gene combinations, standard *Agrobacterium*‐mediated transformation protocols were used. Transformation experiments in tobacco were conducted with *A. tumefaciens* strain GV2260. Kanamycin‐resistant shoots were selected from infected leaf disks on plant regeneration medium supplemented with 50 mg/L kanamycin. Transformation experiments in *Arabidopsis thaliana* ecotype Col‐0 were performed by floral dipping (Clough and Bent, 1998) using *A. tumefaciens* strain GV3101. Primary transgenic plants were identified by BASTA spraying (10 mg/L), and survivors were grown to maturity under standard greenhouse conditions for seed production. The transgenic lines are designated ‘N–’ for *Nicotiana tabacum* and ‘A–’ for *Arabidopsis thaliana*, respectively, followed by the number of the *CPK* transgene(s) they harbour and a consecutive number indicating the transgenic line (e.g. A‐28‐4: transgenic *Arabidopsis* line expressing *CPK28*, line number 4).

### Gene expression analyses

For gene expression analysis, RNA was extracted with the RNeasy Plant Mini Kit (Qiagen) or the NucleoSpin RNA Plant Mini Kit (Macherey & Nagel) according to the manufacturers' instructions and including an on‐column treatment with RNase‐free DNase (Qiagen). Samples of 1 μg RNA were reverse transcribed with SuperscriptIII SuperMix (Invitrogen) according to the manufacturer's instructions. Real‐time quantitative PCR (qRT‐PCR) analysis was performed in a final volume of 10 μL using the Power SYBR Green PCR Master Mix (Applied Biosystems) and following the protocol of the supplier. Reactions were run in a CFX96 system (Bio‐Rad) or a LightCycler 480 instrument (Roche). Expression data for *Arabidopsis* genes were extracted from the eFP Browser (Winter et al., 2007).

### Construction of vectors for plant transformation

To generate vectors for combinatorial transformation, a HindIII/EcoRI fragment containing a new multiple cloning site (MCSnew) followed by a TAG stop codon and the *nos* terminator (T*_nos_*) was introduced into pUC18 (Überlacker and Werr, 1996), yielding plasmid pUC18_MCS_NosT. Subsequently, one of the following promoters was inserted as HindIII/SpeI fragment: P*_SUPERR_*, P*_ACT2_* or P*_UBQ10_*. In the plasmid harbouring P*_UBQ10_*, the *nos* terminator was replaced by the *HSP18.2* terminator cloned as NdeI/EcoRI restriction fragment. The coding sequences of the 15 *CPK* genes were cloned as SpeI/XmaI restriction fragments (except for *CPK29* which was cloned as SpeI/SalI fragment) into these three basic vectors as illustrated in Figure 2a. To enable optimal translation, the sequence AAA was introduced between the 5′ restriction site and the ATG start codon (Joshi *et al*., 1997). The coding regions of the *CPK* genes were amplified from *Arabidopsis* cDNA with primers PS_057 to PS_086 (Table S3).

Vectors for re‐transformation of *CPK28* and *CPK29* were generated by cloning two annealed oligonucleotides (P3687 and P3688; containing XhoI, ClaI, Acc65I and MluI restriction sites) into vector pXCS‐YFP (Feys *et al*., 2005) removing the YFP gene. The *UBIQUITIN10* (*UBQ10*) promoter was amplified with primers P3689/P3690 and inserted into the PmeI/NotI sites of the vector. Additional restriction sites (AgeI, EcoRI, XmaI, NdeI, AatII) were introduced at the proximal end of the promoter by adding the appropriate recognition sequences to the 5' end of oligonucleotide P3690 (Table S3). The terminator for the second expression cassette (*HSP18.2*; AT5g59720) was amplified from *Arabidopsis* genomic DNA with primer pair P3691/P3692, and the resulting fragment was cloned into the AatII and PmeI sites. The CaMV 35S promoter was replaced with the *UBIQUITIN4‐2* promoter amplified from plasmid pPZP221 (Hajdukiewicz *et al*., 1994) with primer pair P3742/P3743 and cloned into the XhoI/AscI restriction sites. To obtain a vector with the 35S promoter in front of both transgene cassettes, the 35S promoter from vector pBI121 was amplified with primer pair P3974/P3975 and introduced as NotI/EcoRI fragment. The *CPK28* coding region was amplified with primers P4762/P4763 from a previously cloned cDNA (Matschi *et al*., 2015). The *CPK29* coding region was amplified with primers P4758/P4759 from *Arabidopsis* cDNA. The coding regions were cloned as EcoRI/XmaI restriction fragments into the above described pXCS derivatives, generating transformation vectors pA‐28 and pA‐29, respectively (Figure 2a). To create vector pA‐28/29, the *CPK29* coding region was amplified with primer pair P4760/4761 and inserted into the KpnI site. An analogous strategy was used to create the expression cassettes in vectors pN‐29 and pN‐28/29 (Figure 3a). To obtain vectors with the *nptII* resistance marker for tobacco transformation, the expression cassettes were amplified with primer pairs P5149/P5152, P5151/P5150 and P5150/P5149 and transferred into the pORE‐E4 plasmid backbone (Coutu *et al*., 2007) as BamHI/XmaI fragments, generating the final transformation vectors pN‐28, pN‐29 and pN‐28/29. All primers are listed in Table S3.

To construct a vector for re‐transformation of *CPK5* into *Nicotiana tabacum*, the coding sequence of *CPK5* (including a C‐terminal StrepII tag) was amplified with specific primers (Table S3) from plasmid pXCSG::*CPK5‐StrepII* (Dubiella *et al*., 2013) and inserted as BamHI/SacI fragment into the binary vector pGreen0179. The vector contains a hygromycin resistance gene for selection of transgenic plants, and the *MAS* promoter to drive expression of the transgene of interest.

### Genotyping of plant lines

Genotyping of *cpk28* and *cpk29* T‐DNA lines was done using the primer pairs recommended by the SALK database (http://signal.salk.edu/tdnaprimers.2.html). To verify transgene presence in combinatorially transformed and retransformed tobacco lines, the same primer pairs were used as for the initial cloning of the coding regions (Table S3). PCR amplification was carried out with GoTaq DNA polymerase (Promega Corp.) and the reaction buffer supplied by the manufacturer. Reaction conditions were chosen according to the manufacturer's instructions.

### Growth conditions and stress tolerance assays

For all physiological measurements (gas exchange, photosynthetic electron transfer, WUE under different watering regimes), wild type and transgenic tobacco lines were grown in a controlled environment chamber under standard conditions (light intensity: 350 µE/m^2^/s, day length: 16 h, day temperature: 22 °C, night temperature: 18 °C, relative humidity: 75% during the day, 70% during the night).

T1 seeds were used for all stress assays. For drought tolerance assays, wild‐type tobacco plants and transgenic plants were grown under standard greenhouse conditions (diurnal cycle: 16 h light at 25 °C and 8 h darkness at 20 °C, average relative humidity: ~65%). In the initial screening, WT and transgenic lines were grown together in a tray, sharing the same soil. The WT control plants were placed as a vertical or a diagonal line separating the transgenic plants. Seeds were germinated on MS medium without sugar after stratification for 3 days at 4 °C. 15 day‐old plants were then transferred to soil and grown for two weeks under standard greenhouse conditions. Pots with individual plants were arranged in a randomized block design. Drought stress was applied by withholding water for 22–28 days (depending on the outside weather conditions), and survival was scored 7 days after rewatering. To assess tobacco growth under water‐limiting conditions, water supply was reduced to 25% (i.e. 25 mL daily), and plants were compared to a well‐watered control group (100 mL daily). For exposure of *Arabidopsis* plants to severe drought stress, transgenic plants and control plants were grown together in trays or, alternatively, in individual pots using a randomized block design. Plants were germinated on peat pellets after 2–4 days stratification at 4 °C, transferred to soil after 10 days and grown under long‐day conditions (16 h light at 22°C, 8 h darkness at 18°C, 60‐65% relative humidity) for 20 days. To induce drought stress, water was withheld for 14 d, followed by re‐watering and scoring of the survivorship after two days. For salt stress experiments, plants were watered with 200 mm NaCl until the soil was saturated and then grown further for one additional week.

For *in vitro* assays, MS medium without added sugar was used for tobacco, except for the BRZ treatments, in which the medium was supplemented with 1 mm sucrose, while for *Arabidopsis*, ½ MS medium with 1% glucose added was used. For stress experiments, the medium was supplemented with either 200 mm NaCl (salt stress) or 35 mm mannitol (osmotic stress; Claeys *et al*., 2014). Seeds were stratified for 3 days at 4°C and then grown under long‐day conditions for 14 days (*Arabidopsis*) or 21 days (tobacco), before fresh weight was measured. For treatments with the brassinosteroid biosynthesis inhibitor BRZ (1 µm), a 3 h light pulse was applied and the Petri dishes with the germinating seeds were kept in the dark for 7 days before hypocotyl length was measured. *In vitro* plants were raised under standard growth conditions in a growth chamber at a light intensity of 100–120 µE/m^2^/s.

### Physiological analyses

Gas exchange measurements were performed as described previously (Schöttler *et al*., 2017). Briefly, a GFS‐3000 open gas exchange system equipped with the LED array unit 3055‐FL as an actinic light source (Heinz Walz GmbH, Effeltrich, Germany) was used to record light‐response curves of CO_2_ assimilation (at 22°C cuvette temperature, 17 500 ppm humidity, and a CO_2_ concentration of 450 ppm). After leaf respiration had been determined in darkness, the actinic light intensity was stepwise increased to 100, 200, 350, 500, 1000 and finally 1500 µE/m^2^/s. Gas exchange was measured at each light intensity until the steady state of transpiration and leaf assimilation was reached.

The intrinsic water‐use efficiency (iWUE) was calculated as the instantaneous ratio between the net CO_2_ assimilation rate (A) and the stomatal conductance to water vapour (gH_2_O). For light intensities close to or below the light compensation point, values were not calculated, to avoid negative values for iWUE.

Chlorophyll‐a fluorescence parameters were measured with a Dual‐PAM‐100 instrument (Heinz Walz GmbH). The maximum quantum efficiency of PSII was determined after 30 min of dark adaptation. Then, the capacity of linear electron transport was determined from light response curves of chlorophyll‐a fluorescence and was subsequently corrected for leaf absorptance. Leaf absorptance was calculated as 100% incident light minus light transmitted through the leaf (%) minus light reflected on the leaf surface (%). Transmittance and reflectance spectra between 400 and 700 nm wavelength were recorded by using an integrating sphere attached to a photometer (V650, Jasco GmbH, Groß‐Umstadt, Germany). The spectral bandwidth was set to 1 nm, and the scanning speed was 200 nm/min. Chlorophyll contents and chlorophyll a:b ratios were measured with a Jasco V‐630 photometer (Jasco GmbH) in 80 % (v/v) acetone (Porra *et al*., 1989).

Stomatal opening was assessed by stomatal aperture measurements. To this end, 2‐week‐old seedlings raised on synthetic medium were transferred to soil and grown under standard greenhouse conditions (19–21 °C, 16/8 h light/dark cycle, 60–65% relative humidity). Leaves from 5‐ to 6‐week‐old plants were sampled, and explants were incubated in stomatal opening buffer (10 mm KCl, 50 µm CaCl_2_, 25 mm MES‐Tris pH 6.5) for 2.5 h under a light intensity of 150 µE/m^2^/s. The explants were then exposed to 0.05% ethanol (control) or 10 µm ABA in 0.05% ethanol for 2 h. Subsequently, epidermal peels were prepared and fixed in lactic acid and images of stomata from the abaxial side of the leaves were taken with the Olympus epifluorescence microscope BX‐51.

## Conflict of interest

The authors declare no conflict of interest.

## Author contributions

P.S., M.A.S., T.R., J.K. and R.B. designed the experiments; P.S., K.P., R.L. and M.A.S. performed the experiments; all authors interpreted and analysed the data; and R.B. and P.S. wrote the paper with input from all other authors.

## Supporting information


**Figure S1** Protein structure and expression patterns of CPK28 and CPK29 from *Arabidopsis*.
**Figure S2** Stress tolerance tests with additional A‐28/29 and A‐28 x A‐29 lines.
**Figure S3** The gene combination *CPK28* and *CPK29* has no impact on plant biomass production under control conditions in *Arabidopsis*.
**Figure S4** The CPK28/29 module confers tolerance to salt stress.
**Figure S5** Expression of *CPK28* and *CPK29* has no impact on gas exchange in control conditions.
**Figure S6** Expression of *CPK28* and *CPK29* has no impact on stomatal responses to ABA.
**Figure S7** Expression of *CPK28* and *CPK29* does not alter the stomatal index.
**Figure S8** Expression of *CPK28* and *CPK29* has no impact on photosynthetic parameters measured in plants grown under well‐watered conditions *versus* plants exposed to mild water‐limited conditions.
**Figure S9** Expression of *CPK28* and *CPK29* in wild‐type and transgenic *Arabidopsis* seedlings as determined by qRT‐PCR analyses.
**Figure S10** Expression of *CPK28* and *CPK29* in wild‐type and transgenic tobacco seedlings as determined by qRT‐PCR analyses.
**Figure S11** Expression of *CPK5* in wild‐type and transgenic tobacco seedlings as determined by qRT‐PCR analysis.
**Table S1** Representation of *CPK3* and *CPK29* in combinatorially transformed tobacco lines exhibiting drought tolerance.
**Table S2** Genotyping of drought‐tolerant segregants from crosses of hemizygous N‐28 with N‐29 lines.
**Table S3** List of oligonucleotides used in this study.
