# Supplementary material for: Improving plant drought tolerance and growth under water limitation through combinatorial engineering of signalling networks
Source: Plant Biotechnol J. 2020 Jul 26;19(1):74–86. doi: 10.1111/pbi.13441 (PMC7769235; doi:10.1111/pbi.13441)
Supplement: Supplementary file 1 — Figure S1 Protein structure and expression patterns of CPK28 and CPK29 from Arabidopsis. Figure S2 Stress tolerance tests with additional A‐28/29 and A‐28 x A‐29 lines. Figure S3 The gene combination CPK28 and CPK29 has no impact on plant biomass production under control conditions in Arabidopsis. Figure S4 The CPK28/29 module confers tolerance to salt stress. Figure S5 Expression of CPK28 and CPK29 has no impact on gas exchange in control conditions. Figure S6 Expression of CPK28 and CPK29 has no impact on stomatal responses to ABA. Figure S7 Expression of CPK28 and CPK29 does not alter the stomatal index. Figure S8 Expression of CPK28 and CPK29 has no impact on photosynthetic parameters measured in plants grown under well‐watered conditions versus plants exposed to mild water‐limited conditions. Figure S9 Expression of CPK28 and CPK29 in wild‐type and transgenic Arabidopsis seedlings as determined by qRT‐PCR analyses. Figure S10 Expression of CPK28 and CPK29 in wild‐type and transgenic tobacco seedlings as determined by qRT‐PCR analyses. Figure S11 Expression of CPK5 in wild‐type and transgenic tobacco seedlings as determined by qRT‐PCR analysis. Table S1 Representation of CPK3 and CPK29 in combinatorially transformed tobacco lines exhibiting drought tolerance. Table S2 Genotyping of drought‐tolerant segregants from crosses of hemizygous N‐28 with N‐29 lines. Table S3 List of oligonucleotides used in this study. [file PBI-19-74-s001.pdf]

Supporting information

A

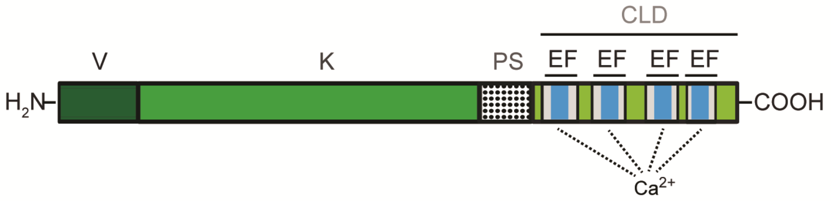
CLD
V
K
PS
EF EF EF EF
H2N
COOH
Ca2+

B

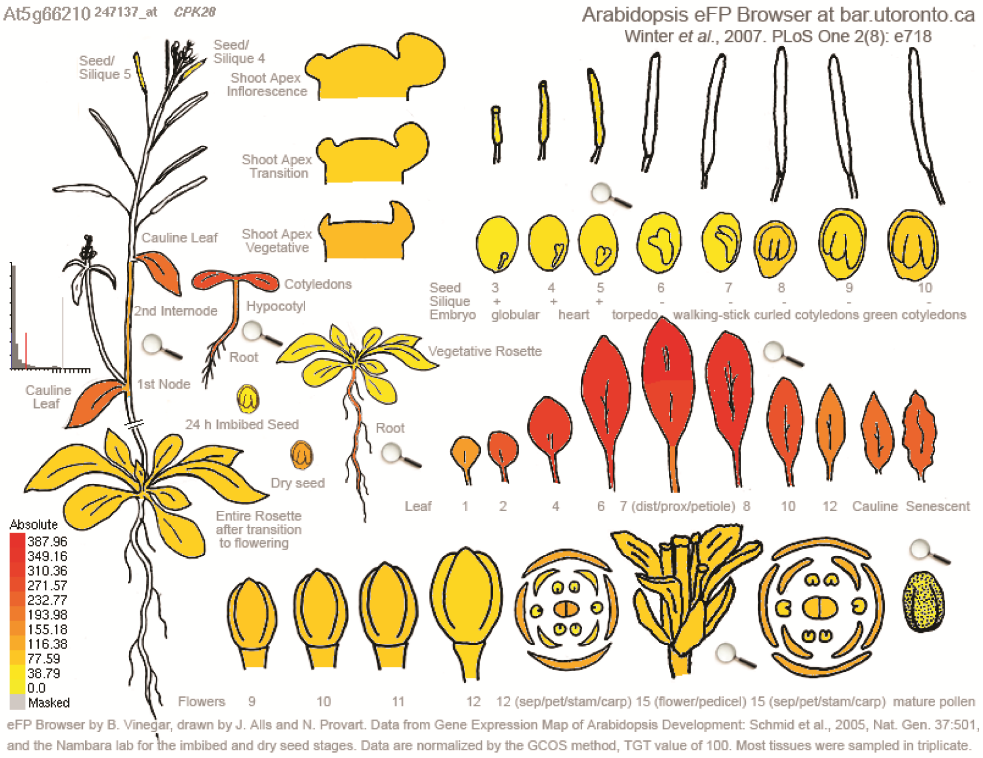
At5g66210 247137_at CPK28
Arabidopsis eFP Browser at bar.utoronto.ca
Winter et al., 2007. PLoS One 2(8): e718
Seed/ Silique 5
Seed/ Silique 4
Shoot Apex Inflorescence
Shoot Apex Transition
Shoot Apex Vegetative
Cauline Leaf
Cotyledons
Hypocotyl
2nd Internode
Root
1st Node
Cauline Leaf
24 h Imbibed Seed
Vegetative Rosette
Root
Dry seed
Entire Rosette after transition to flowering
Seed 3 4 5 6 7 8 9 10
Silique + + + - - - - -
Embryo globular heart torpedo walking-stick curled cotyledons green cotyledons
Leaf 1 2 4 6 7 (dist/prox/petiole) 8 10 12 Cauline Senescent
Absolute
387.96
349.16
310.36
271.57
232.77
193.98
155.18
116.38
77.59
38.79
0.0
Masked
Flowers 9 10 11 12 12 (sep/pet/stam/carp) 15 (flower/pedicel) 15 (sep/pet/stam/carp) mature pollen
eFP Browser by B. Vinegar, drawn by J. Alls and N. Provart. Data from Gene Expression Map of Arabidopsis Development: Schmid et al., 2005, Nat. Gen. 37:501, and the Nambara lab for the imbibed and dry seed stages. Data are normalized by the GCOS method, TGT value of 100. Most tissues were sampled in triplicate.

C

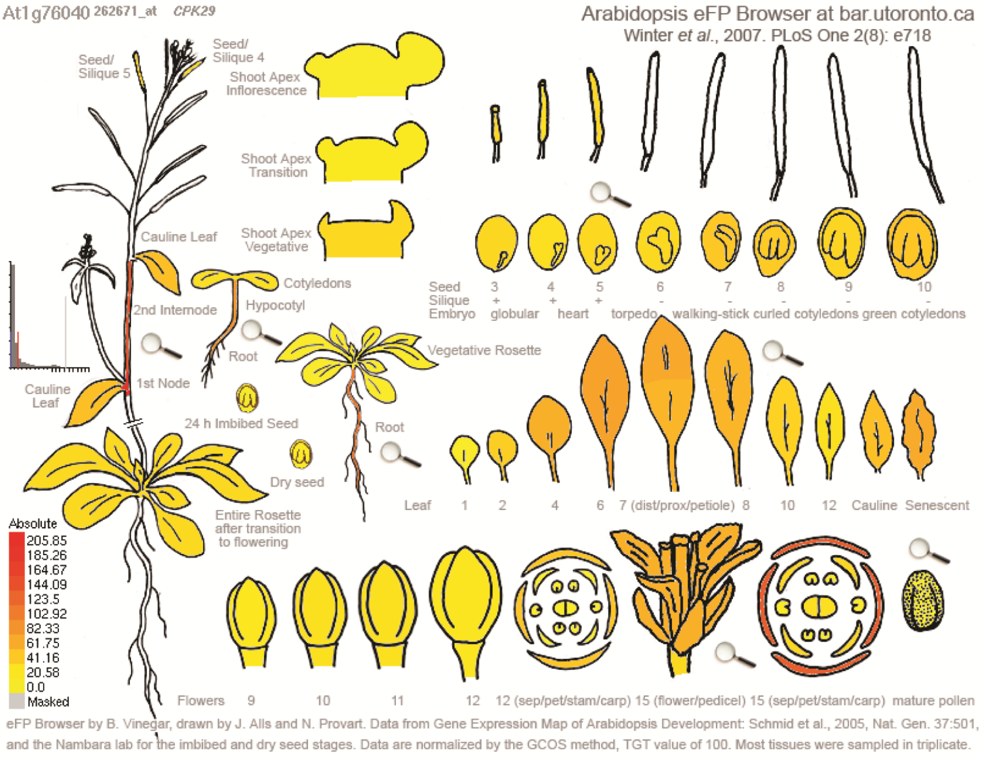
At1g76040 262671_at CPK29
Arabidopsis eFP Browser at bar.utoronto.ca
Winter et al., 2007. PLoS One 2(8): e718
Seed/ Silique 5
Seed/ Silique 4
Shoot Apex Inflorescence
Shoot Apex Transition
Shoot Apex Vegetative
Cauline Leaf
Cotyledons
Hypocotyl
2nd Internode
Root
1st Node
Cauline Leaf
24 h Imbibed Seed
Vegetative Rosette
Root
Dry seed
Entire Rosette after transition to flowering
Seed 3 4 5 6 7 8 9 10
Silique + + + - - - - -
Embryo globular heart torpedo walking-stick curled cotyledons green cotyledons
Leaf 1 2 4 6 7 (dist/prox/petiole) 8 10 12 Cauline Senescent
Absolute
205.85
185.26
164.67
144.09
123.5
102.92
82.33
61.75
41.16
20.58
0.0
Masked
Flowers 9 10 11 12 12 (sep/pet/stam/carp) 15 (flower/pedicel) 15 (sep/pet/stam/carp) mature pollen
eFP Browser by B. Vinegar, drawn by J. Alls and N. Provart. Data from Gene Expression Map of Arabidopsis Development: Schmid et al., 2005, Nat. Gen. 37:501, and the Nambara lab for the imbibed and dry seed stages. Data are normalized by the GCOS method, TGT value of 100. Most tissues were sampled in triplicate.

Figure S1 Protein structure and expression patterns of CPK28 and CPK29 from *Arabidopsis*. (A) Schematic representation of the domain organization of CPK28 and CPK29. The proteins contain the CPK-typical variable (V), kinase (K), pseudosubstrate (PS) and calmodulin-like (CLD) domains. The CLD harbors several Ca^{2+}-binding EF-hand motifs. (B) Expression pattern of *CPK28*. (C) Expression pattern of *CPK29*. Data were extracted from the eFP Browser (Winter et al., 2007). The expression profiles of the two genes show some overlap, but *CPK29* expression in leaves of all developmental stages is substantially lower than *CPK28* expression.

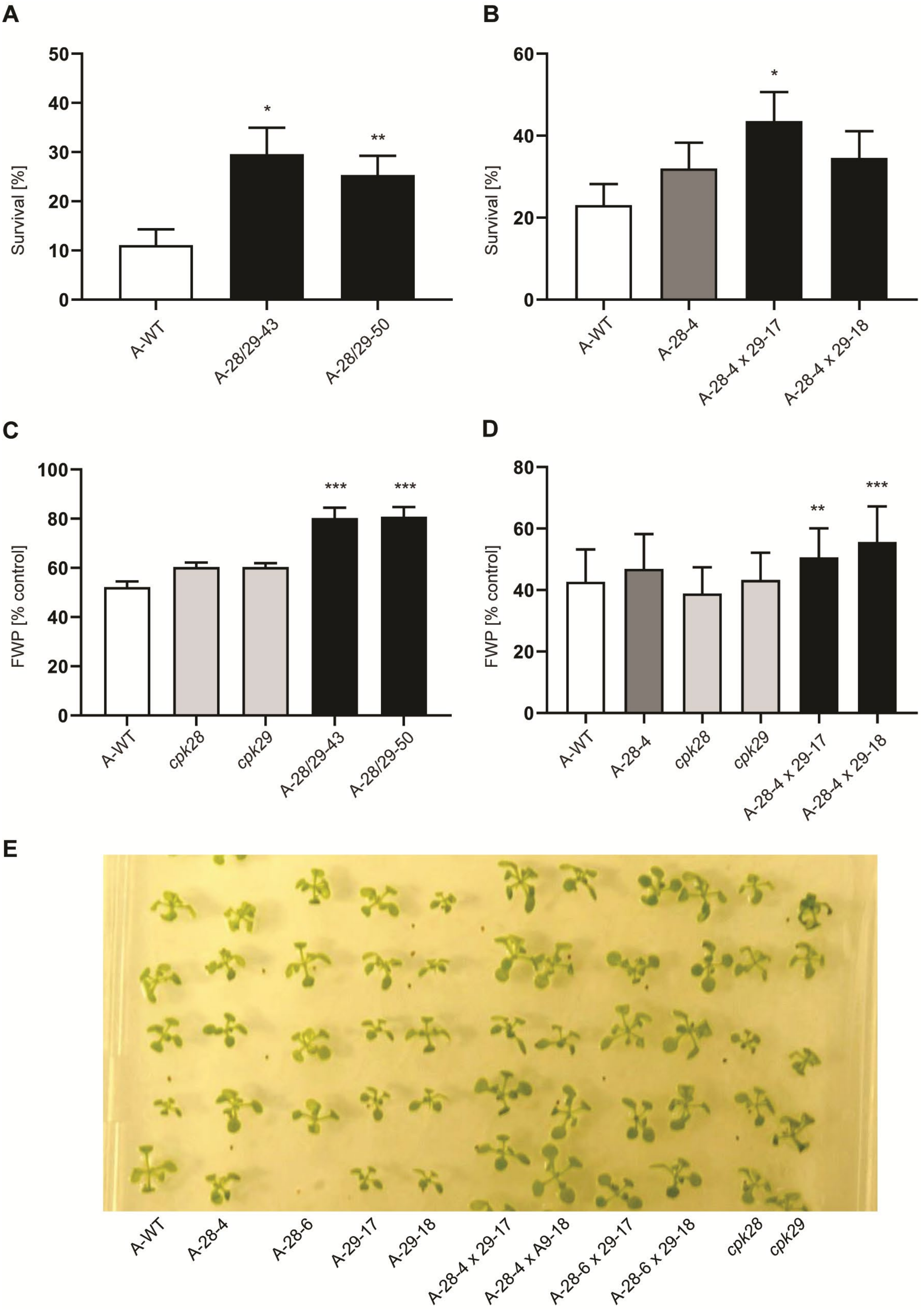
A
50
40
30
20
10
0
Survival [%]
*
**
A-WT
A-28/29-43
A-28/29-50
B
60
40
20
0
Survival [%]
*
A-WT
A-28-4
A-28-4 x 29-17
A-28-4 x 29-18
C
100
80
60
40
20
0
FWP [% control]
***
***
A-WT
cpk28
cpk29
A-28/29-43
A-28/29-50
D
80
60
40
20
0
FWP [% control]
**
***
A-WT
A-28-4
cpk28
cpk29
A-28-4 x 29-17
A-28-4 x 29-18
E
A-WT
A-28-4
A-28-6
A-29-17
A-29-18
A-28-4 x 29-17
A-28-4 x A9-18
A-28-6 x 29-17
A-28-6 x 29-18
cpk28
cpk29

Figure S2 Stress tolerance tests with additional A-28/29 and A-28 x A-29 lines. (A) Increased survival rate of additional transgenic lines produced by transformation with vector pA-28/29 (cf. Figure 2). (B) Enhanced survival of transgenic lines obtained from crosses of A-28 with A-29 lines (A-28 x 29). (C) Increased biomass production of A-28/29 lines grown under mild osmotic stress (35 mM mannitol). (D) Increased biomass production (FWP, fresh weight per plant) of A-28 x 29 lines grown under mild osmotic stress. Asterisks indicate statistically significant differences between transgenic lines and the wild type as determined by a two-tailed unpaired t-test (* = $p<0.05$, ** = $p<0.01$, *** =$p<0.001$). All data are presented as means ± SD from three independent experiments (A: n=9 biological replicates, with 3 plants per line in each replicate; B: n=26 biological replicates, with 3 plants per line in each replicate; C: n=4-12 biological replicates, with 10 plants per line and condition in each replicate; D: n=10 biological replicates for the 2 mutant lines and n=20 for all other lines, with 10 plants per line and condition in each replicate). (E) Representative image of an osmotic stress assay (in the presence of 35 mM mannitol) showing improved growth in the progeny of all of the four different A-28 x A-29 crosses. For comparison, *cpk28* and *cpk29* knock-out mutants were also included.

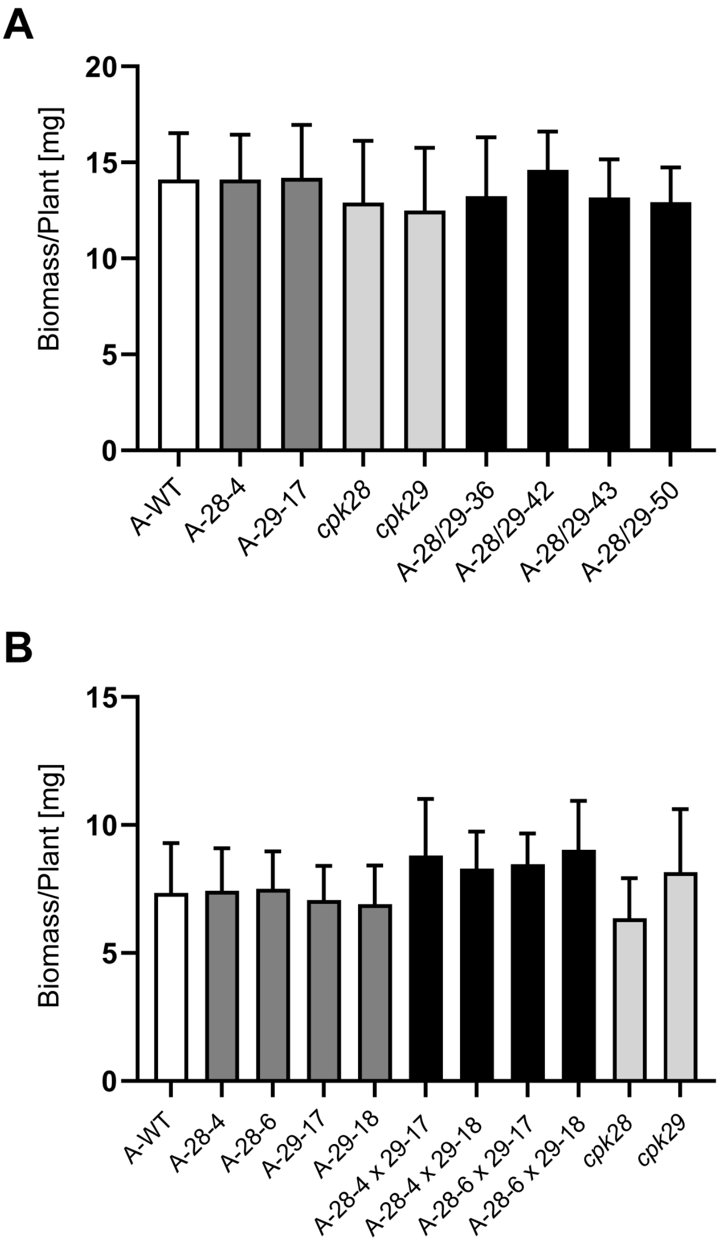

Figure S3 The gene combination *CPK28* and *CPK29* has no impact on plant biomass production under control conditions in *Arabidopsis.* (A) Biomass under control conditions of the plants used in Figure 2F and Figure S2C. (B) Biomass under control conditions of the plants used in Figure 2G and Figure S2D. No statistically significant differences are found by one-way ANOVA.

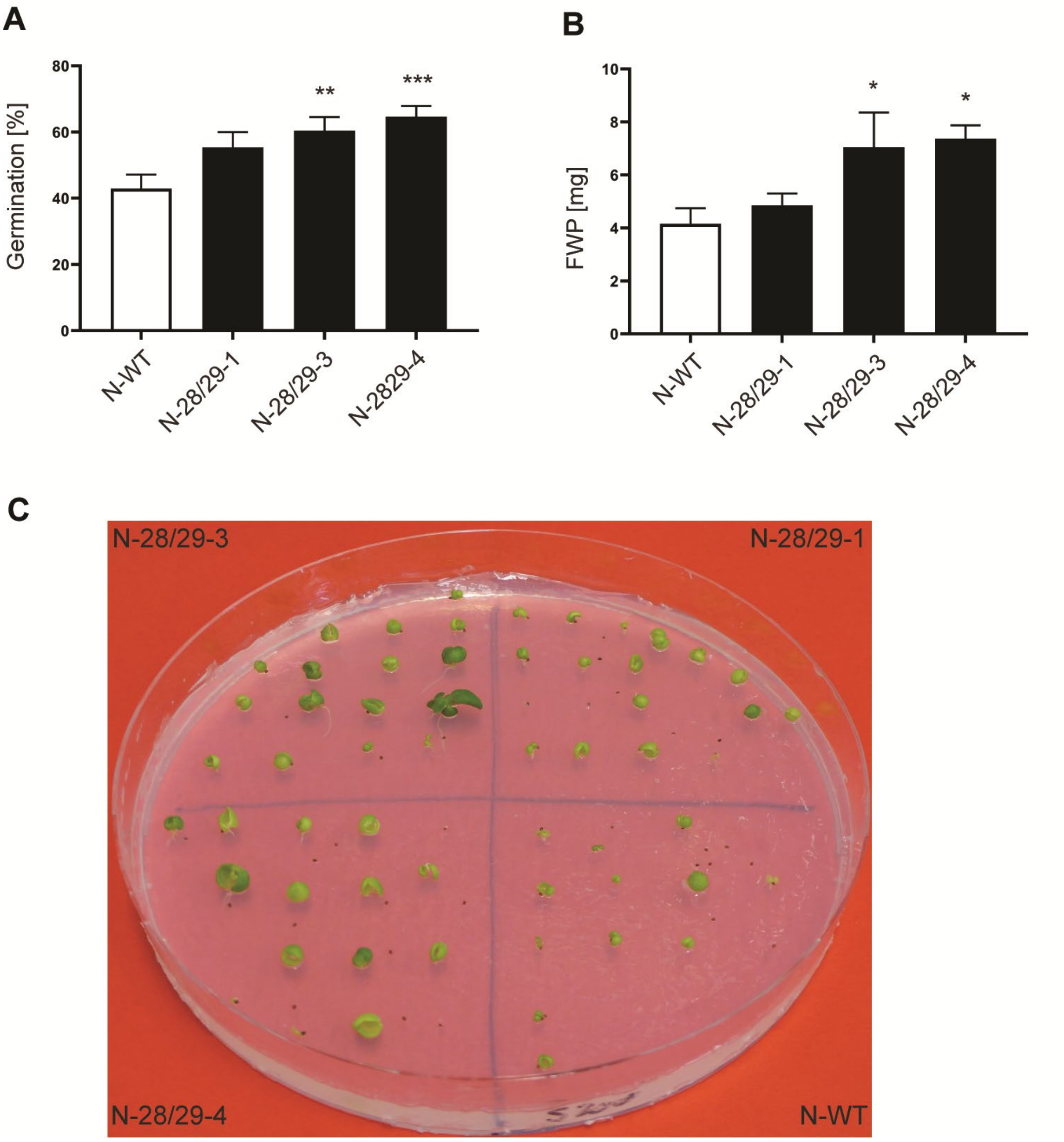

Figure S4 The CPK28/29 module confers tolerance to salt stress. (A) Improved seed germination of the N28/29-3 and N-28/29-4 lines compared to the wild type in the presence of 200 mM NaCl. Germination rates were scored after 21 days. (B) Enhanced biomass production of lines N-28/29-3 and N-28/29-4 compared to the wild type after 28 days of growth in the presence of 200 mM NaCl. (C) Representative picture of a germination assay on medium with 200 mM NaCl after 21 days. Asterisks indicate statistically significant differences between transgenic lines and the wild type as determined by two-tailed unpaired t-tests (* = $p<0.05$, ** = $p<0.01$, *** = $p<0.001$).

All data are presented as means ± SD from three independent experiments (A: n=28 biological replicates, each with 16 seeds per line; B: n=10 biological replicates, each with up to 16 seedlings per line).

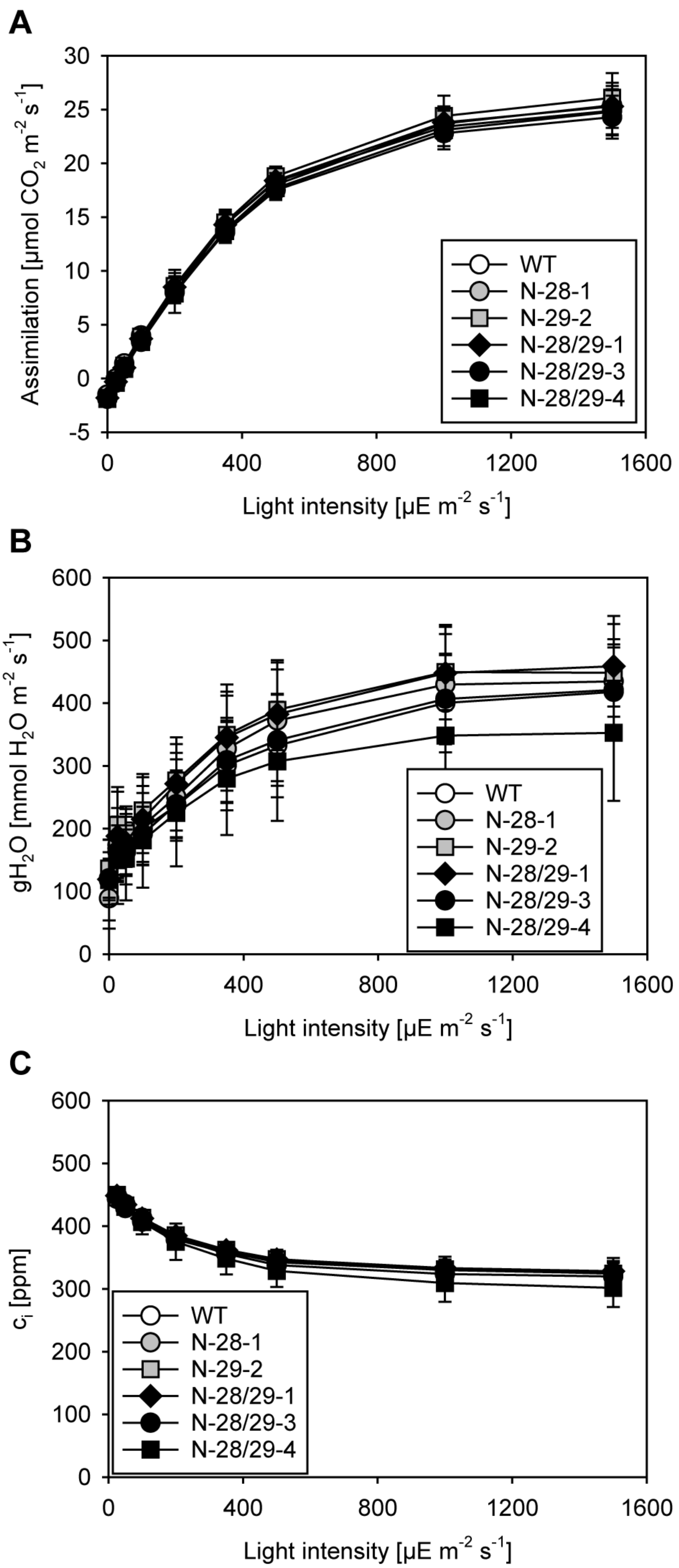
A
Assimilation [µmol CO2 m-2 s-1]
Light intensity [µE m-2 s-1]
WT
N-28-1
N-29-2
N-28/29-1
N-28/29-3
N-28/29-4
B
gH2O [mmol H2O m-2 s-1]
Light intensity [µE m-2 s-1]
WT
N-28-1
N-29-2
N-28/29-1
N-28/29-3
N-28/29-4
C
ci [ppm]
Light intensity [µE m-2 s-1]
WT
N-28-1
N-29-2
N-28/29-1
N-28/29-3
N-28/29-4

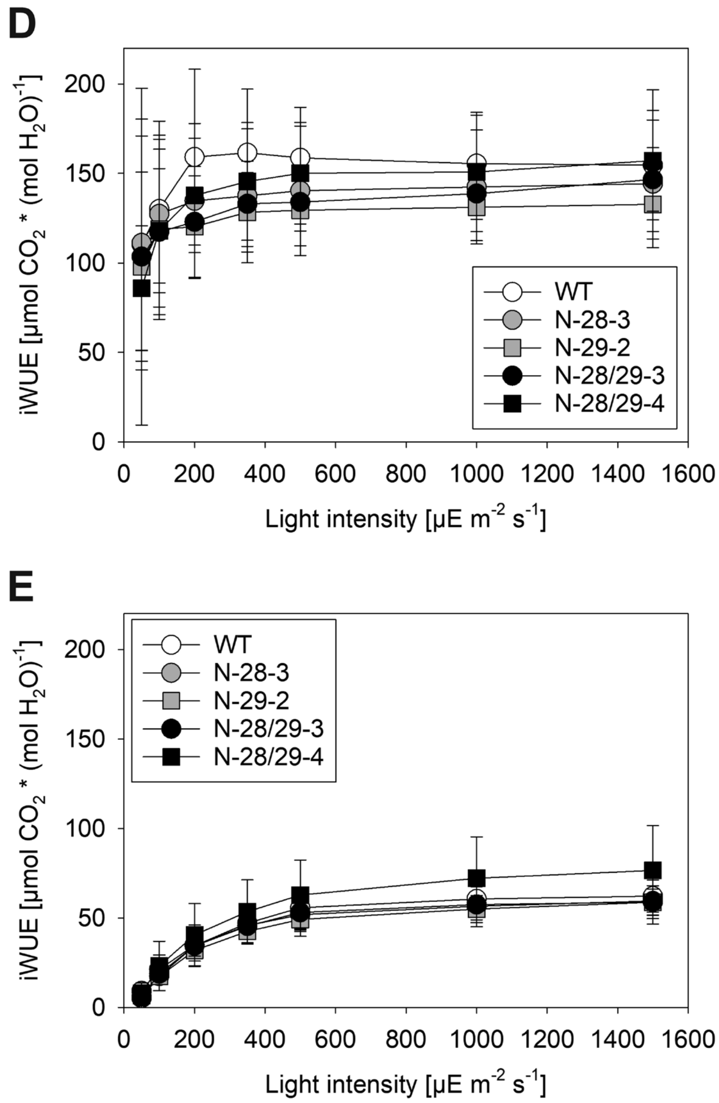

Figure S5 Expression of *CPK28* and *CPK29* has no impact on gas exchange in control conditions. (A) Measurement of photosynthetic assimilation capacity. (B) Measurement of stomatal conductance. (C) Measurement of the internal CO_2 concentration in leaves. WT: wild-type tobacco plants. No statistically significant differences can be detected between lines grown under the same conditions (determined by one-way ANOVA). All data are presented as means ± SD from two independent experiments (n=11, 10, 9, 6, 10 and 10 biological replicates for WT, N-28-1, N-29-2, N-28/29-1, N-28/29-3 and N-28/29-4, respectively). (D,E) Intrinsic water-use efficiency (iWUE) under water-limited conditions (D; cf. Figure 4), and under control conditions (E). For details, see Experimental procedures.

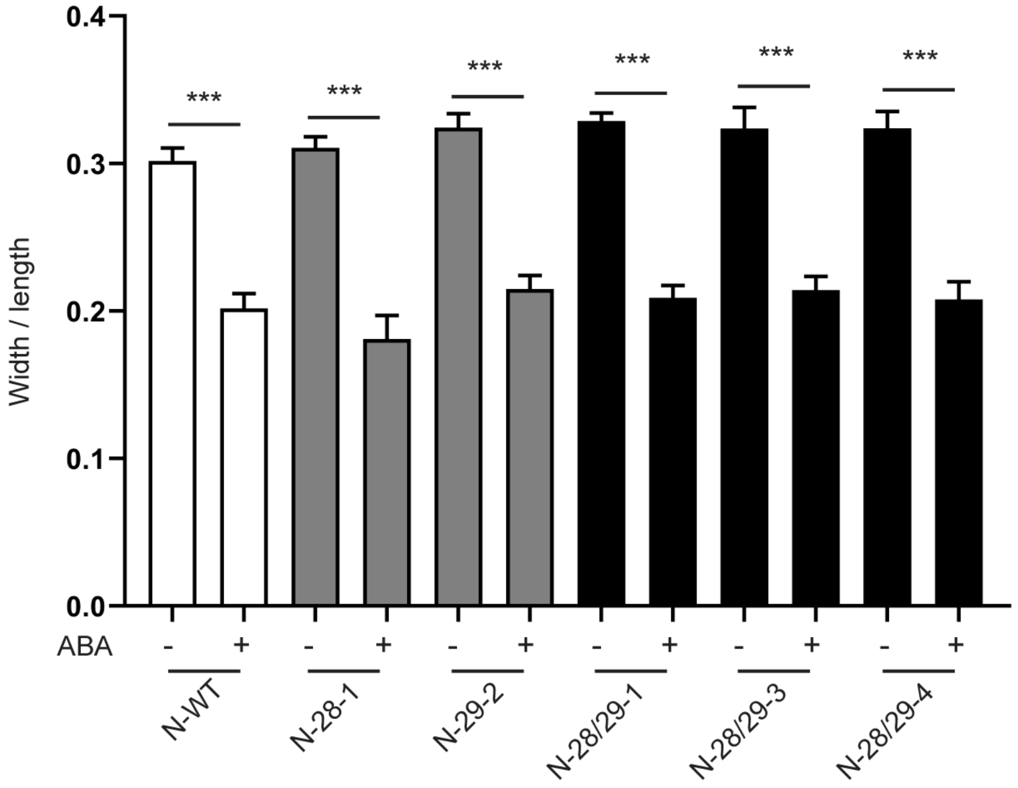

Figure S6 Expression of *CPK28* and *CPK29* has no impact on stomatal responses to ABA. Stomatal width/length ratios were determined after incubation in stomatal opening solution for two hours and an additional two-hour incubation with or without 10 µM ABA. The asterisks indicate statistically significant differences between the lines with and without ABA treatment as determined by one-way ANOVA followed by Tukey´s multiple test (*** = $p<0.001$). All data are presented as means ± SD of n=19/31, 18/21, 14/23, 24/34, 16/19 and 14/18 stomata measured without/ with ABA from N-WT, N-28-1, N-29-2, N28/29-1, N-28/29-3 and N28/29-4, respectively.

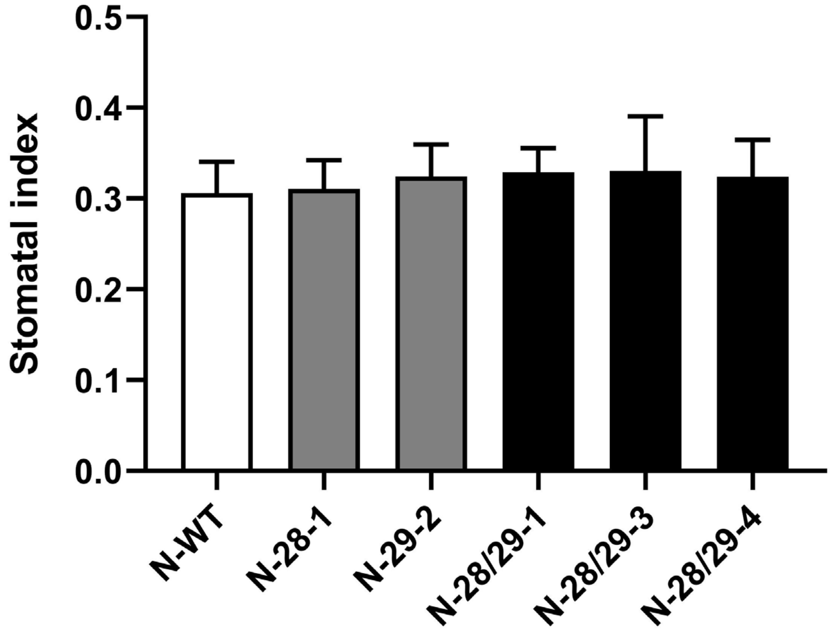

Figure S7 Expression of *CPK28* and *CPK29* does not alter the stomatal index. Guard cell number per total cell number was determined based on microscopic images of non-stressed plants (cf. Figure S6). At least 30 cells per image were counted. Data are presented as means ± SD of n=19, 18, 14, 24, 16 and 14 images from N-WT, N-28-1, N-29-2, N28/29-1, N-28/29-3 and N28/29-4, respectively.

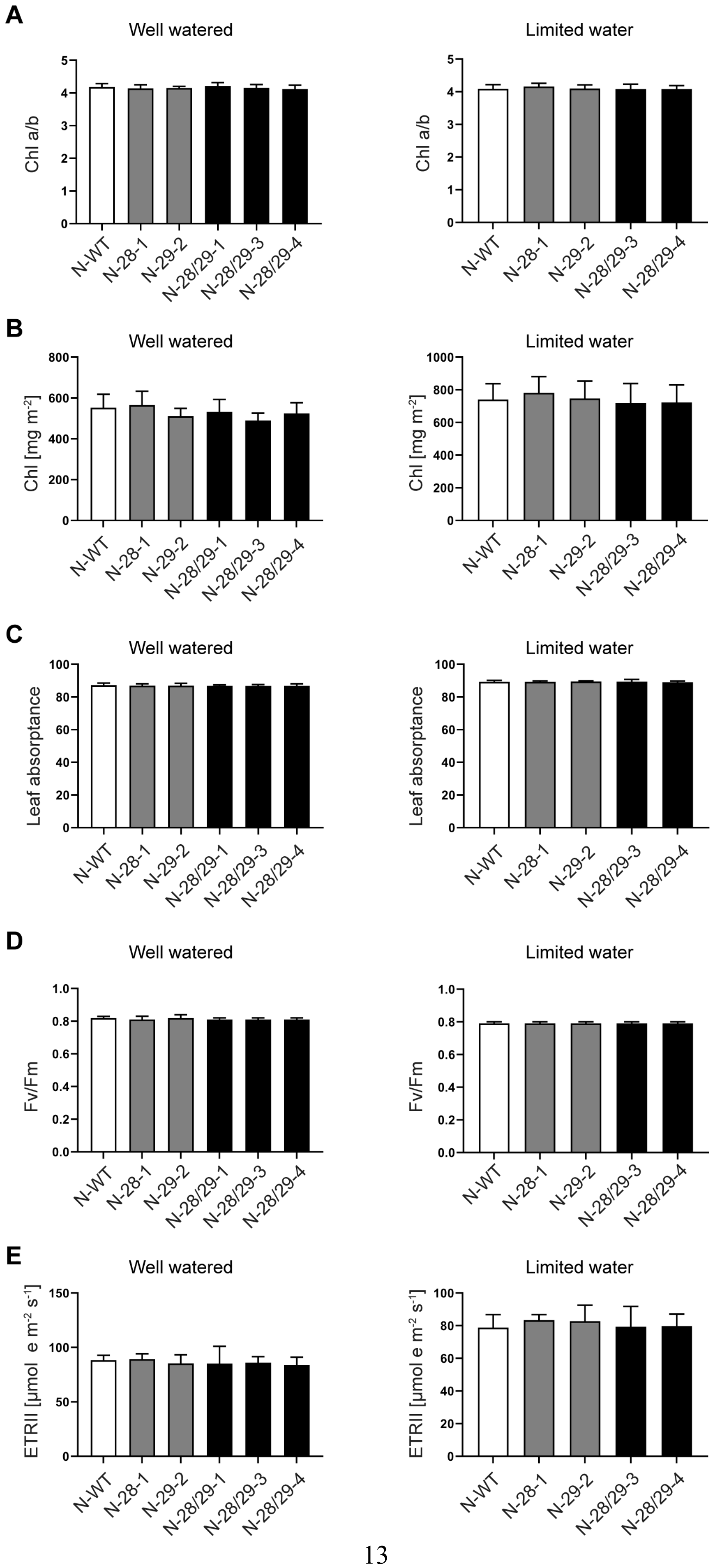
A
Well watered
Limited water
Chl a/b
0
1
2
3
4
5
N-WT
N-28-1
N-29-2
N-28/29-1
N-28/29-3
N-28/29-4
B
Well watered
Limited water
Chl [mg m-2]
0
200
400
600
800
1000
C
Well watered
Limited water
Leaf absorptance
0
20
40
60
80
100
D
Well watered
Limited water
Fv/Fm
0.0
0.2
0.4
0.6
0.8
1.0
E
Well watered
Limited water
ETRII [µmol e m-2 s-1]
0
50
100
150
0
20
40
60
80
100

Figure S8 Expression of *CPK28* and *CPK29* has no impact on photosynthetic parameters measured in plants grown under well-watered conditions *versus* plants exposed to mild water-limited conditions. (A) Chlorophyll a:b ratio. (B) Total chlorophyll content per leaf area. (C) Leaf absorptance. (D) Maximum quantum efficiency of photosystem II in the dark-adapted state (F_v/F_m). (E) Electron transport capacity of PSII (ETRII). No statistically significant differences between the lines grown in the same conditions were detectable (determined by one-way ANOVA). All data are presented as means ± SD from two independent experiments (n=11, 10, 9, 6, 10 and 10 biological replicates in control conditions for N-WT, N-28-1, N-29-2, N28/29-1, N28/29-3 and N-28/29-4, respectively; and n=8, 8, 8, 8 and 7 biological replicates in water-limiting conditions for N-WT, N-28-1, N-29-2, N28/29-3 and N-28/29-4, respectively).

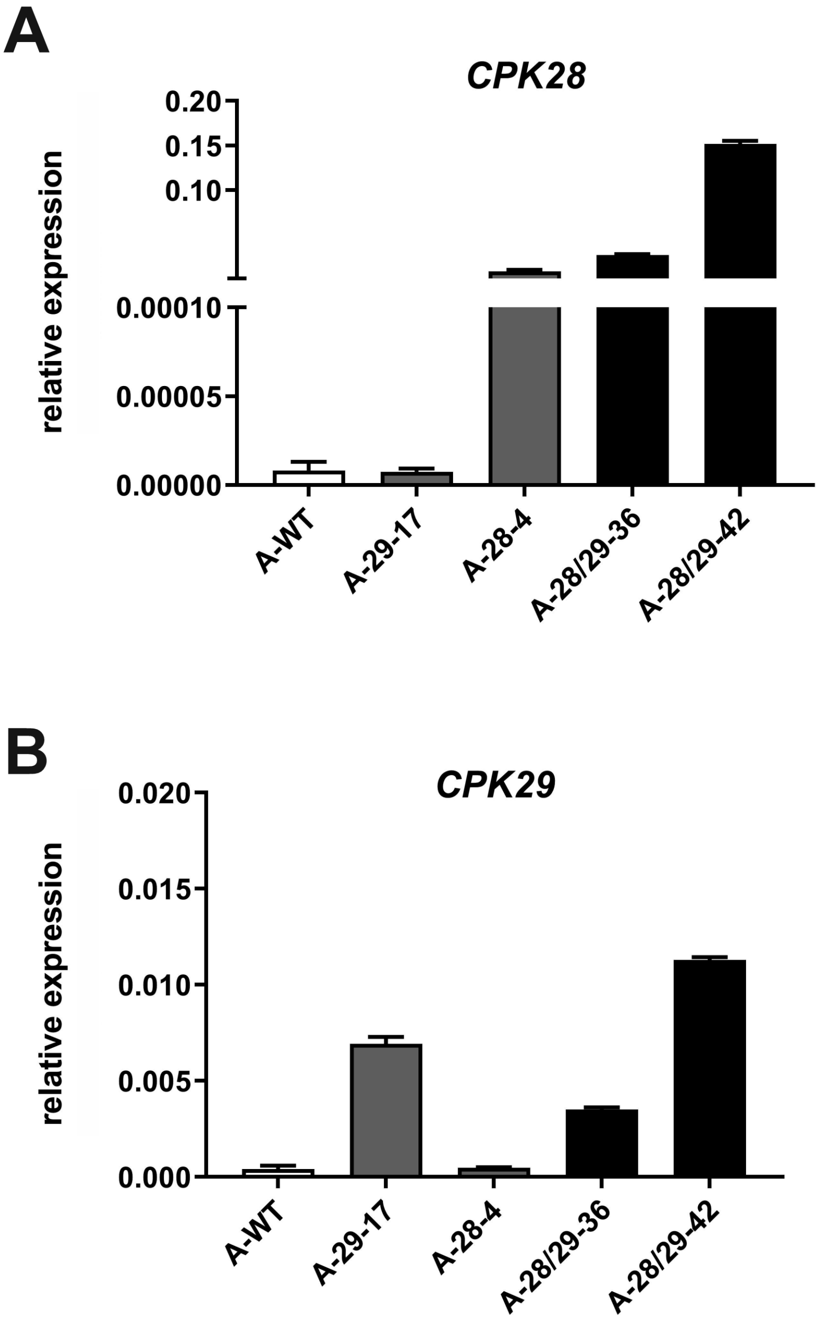

Figure S9 Expression of *CPK28* (A) and *CPK29* (B) in wild-type and transgenic *Arabidopsis* seedlings as determined by qRT-PCR analyses. Three biological replicates were measured and relative expression levels are shown (using *ACT2* as reference gene). Data are presented as means ± SD.

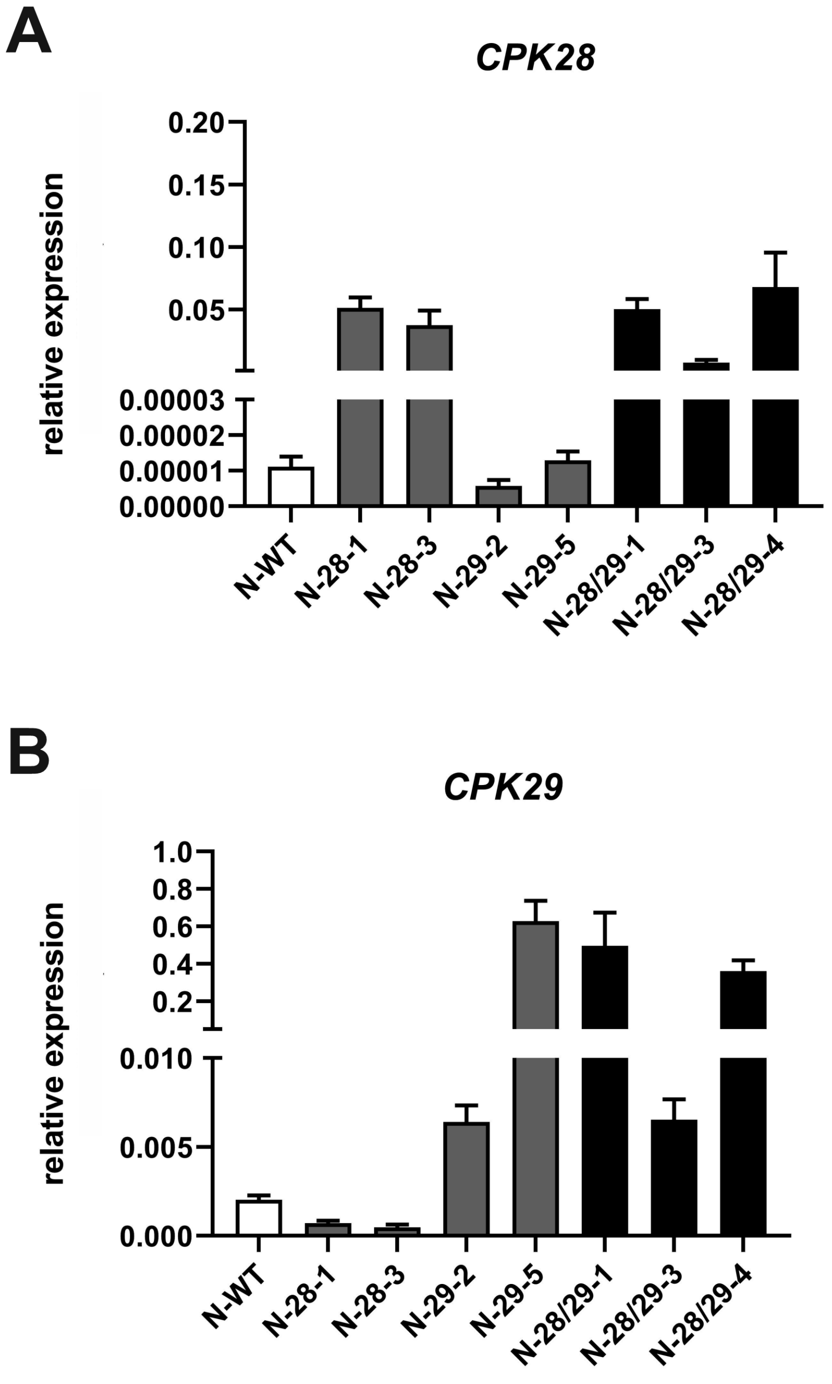

Figure S10 Expression of *CPK28* (A) and *CPK29* (B) in wild-type and transgenic tobacco seedlings as determined by qRT-PCR analyses. Six biological replicates were measured and relative expression levels are shown (using *UBQ* as reference gene). Data are presented as means ± SD.

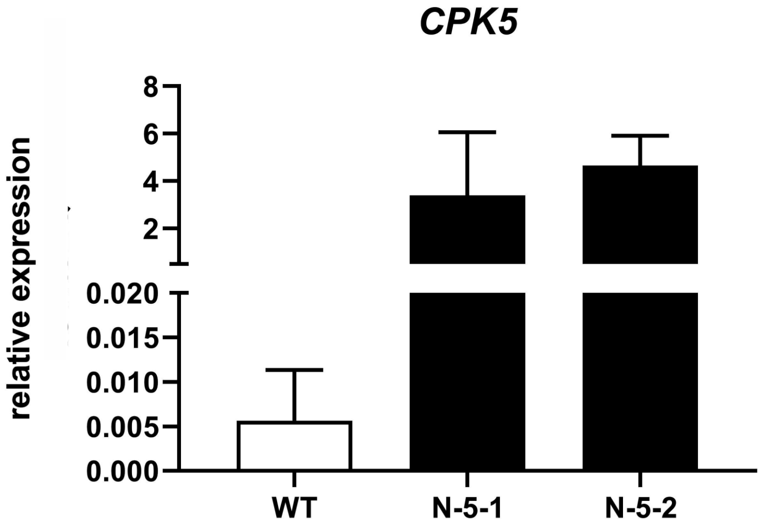

Figure S11 Expression of *CPK5* in wild-type and transgenic tobacco seedlings as determined by qRT-PCR analysis. Three biological replicates were measured and relative expression levels are shown (using *UBQ* as reference gene). Data are presented as means ± SD.

Table S1 Representation of *CPK3* and *CPK29* in combinatorially transformed tobacco lines exhibiting drought tolerance.

Gene	Presence in all lines	Presence in tolerant lines	Expected random frequency	Detected frequency in tolerant lines
CPK3 AT4G23650	20 / 103	4 / 9	19.5%	44.4%
CPK29 AT1G76040	23 / 103	5 / 9	22.3%	55.5%

Table S2 Genotyping of drought-tolerant segregants from crosses of hemizygous N-28 with N-29 lines. **** : <0.0001; *** : <0.001; ** : <0.01; ns: not significant.

**Cross**	**Offspring tested**	**Survivors**	**Both alleles expected [%]**	**Both alleles found [%]**	**Chi-square test**
N-28-1 x N-29-2	109	13	25	46	****
N-28-3 x N-29-2	121	17	25	41	**
N-28-9 x N-29-5	58	14	25	43	***
N-28-9 x N-29-2	52	12	25	33	ns

Table S3 List of oligonucleotides used in this study.

Primer name	DNA target	Sequence 5´- 3´
PS_057	CPK1-fw	TTTTACTAGTAAAATGGGTAATACTTGTGTTGGAC
PS_058	CPK1-rev	AAAACCCGGGCTAGAGTTTAAGAGCAATGCTAAAG
PS_059	CPK3-fw	TTTTACTAGTAAAATGGGCCACAGACACAGC
PS_060	CPK3-rev	AAAACCCGGGTCACATTCTGCGTCGGTTTG
PS_061	CPK4-fw	TTTTACTAGTAAAATGGAGAAACCAAACCCTAGAA
PS_062	CPK4-rev	AAAACCCGGGTTACTTTGGTGAATCATCAGATTTAG
PS_063	CPK5-fw	TTTTACTAGTAAAATGGGCAATTCTTGCCGTGG
PS_064	CPK5-rev	AAAACCCGGGCTACGCGTCTCTCATGCTAATG
PS_065	CPK6-fw	TTTTACTAGTAAAATGGGCAATTCATGTCGTGG
PS_066	CPK6-rev	TTTCCCGGGCTACACATCTCTCATGC
PS_067	CPK8-fw	TTTTACTAGTAAAATGGGAAATTGTTGTGCGAGC
PS_068	CPK8-rev	AAAAGTCGACTTAATTTTCGCCTTCTAATTGCAAT
PS_069	CPK9-fw	TTTTACTAGTAAAATGGGAAATTGTTTTGCCAAGAAC
PS_070	CPK9-rev	AAAACCCGGGCTAGAACAGCCGAGGTTGTT
PS_071	CPK10-fw	TTTTACTAGTAAAATGGGTAACTGTAACGCCTG
PS_072	CPK10-rev	AAAACCCGGGTTAAACAGGAACAGTTTGTCCAGTG
PS_073	CPK11-fw	TTTTACTAGTAAAATGGAGACGAAGCCAAACCC
PS_074	CPK11-rev	AAAACCCGGGTCAGTCATCAGATTTTTCACCATC
PS_075	CPK15-fw	TTTTACTAGTAAAATGGGTTGCTTTAGCAGCAAAC
PS_076	CPK15-rev	AAAACCCGGGCTAGTTTAAGTCAGCAACTCTTTTG
PS_077	CPK21-fw	TTTTACTAGTAAAATGGGTTGCTTCAGCAGTAAAC
PS_078	CPK21-rev	AAAACCCGGGTCAATGGAATGGAAGCAGTTTC
PS_079	CPK28-fw	TTTTACTAGTAAAATGGGTGTCTGTTTCTCCGC
PS_080	CPK28-rev	AAAACCCGGGCTATCGAAGATTCCTGTGACC

PS_081	CPK29-fw	TTTTACTAGTAAAATGCTTCAAAACCAACATAAAACG ACC
PS_082	CPK29-rev	AAAACCCGGGTCATCTGATCAGCTTTGGATCTGAATC
PS_083	CPK30-fw	TTTTACTAGTAAAATGGGTAATTGTATCGCCTGC
PS_084	CPK30-rev	AAAACCCGGGTCAAACTGCAATAGATTGTCCAG
PS_085	CPK32-fw	TTTTACTAGTAAAATGGGTAATTGTTGCGGAACAG
PS_086	CPK32-rev	AAAACCCGGGTCATCTTGTATCACCATTGACCTG
P3687	pXCS_TALEN_1-fw	GACCTTGCTCGAGTAATCGATGCGGTACCTCACGCGTA CGAGCT
P3688	pXCS_TALEN_1-rev	CGTACGCGTGAGGTACCGCATCGATTACTCGAGCAAG
P3689	UBQ_NotI-fw	TAAGCGGCCGCGTCGACGAGTCAGTAATAA
P3690	UBQ_PmeI-rev	TAGTTTAAACGACGTCCATATGCCCGGGACCGGTGAA TTCCTGTTAATCAGAAAAACTCAG
P3691	Term_AatII-fw	ATCGACGTCTATATGAAGATGAAGATGAAA
P3692	Term_PmeI-rev	ATAGTTTAAACCTTATCTTTAATCATATTCC
P3742	pcUBQ-sense	TATGGCGCGCCAAAAATTACGGATATGAATATAGGC
P3743	pcUBQ-antisense	ATAAGGTCCTGCTGCACATACATAACATATCA
P3974	35S_NotI_sense	TAAGCGGCCGCAGATTAGCCTTTTCAATTTCAGAAAG AA
P3975	35S_EcoRI_antisense	ATAGAATTCCGTGTTCTCTCCAAATGAAATG
P4762	CPK28_EcoRI-fw	GAATTCATGGGTGTCTGTTTCTCCG
P4763	CPK28_XmaI-rev	CCCGGGCTATCGAAGATTCCTGTGACC
P4758	CPK29_EcoRI-fw	GAATTCATGCTTCAAAACCAACATAA
P4759	CPK29_XmaI-rev	CCCGGGTCATCTGATCAGCTTTGGATC
P4760	CPK29_KpnI_pcUBQ-fw	GGTACCATGCTTCAAAACCAACATAA
P4761	CPK29_KpnI_pcUBQ-rev	GGTACCTCATCTGATCAGCTTTGGATC
P5149	CPK28_XmaI-rev	CCCGGGCTATCGAAGATTCCTGTGA
P5152	AtUBQ-fw	GGATCCGGCCGCGTCGACGAGTCA
P5151	CPK29_XmaI-rev	CCCGGGGTACCTCATCTGATCAGCTTT
P5150	35S_BamHI-fw	GGATCCGGTCTCAGAAGACCAGAGG
P2372	AtCPK5 BamH1 fw	AAGGATCCATGGGCAATTCTTGCCGTG
P2373	StrepII tag SacI rev	AAGAGCTCTTATTTTTCAAATTGAGGATGAGAC
P2427	qRT CPK28f	CGATACCAAGGCCTCTACCA

P2428	qRT CPK28r	GCCGATGGTGTAGTGATCGT
P5095	qRT CPK29f	ATGGAAGCTGCTGATGTGG
P5096	qRT CPK29r	ATGACGATGCATTGTTGCAG
P3585	qRT CPK5f	TAACAGCGGGGCAATCACAT
P3586	qRT CPK5r	GTCTACATCAGCCGCATCCA
